# Key concepts and reporting recommendations for mapping reviews: A scoping review of 68 guidance and methodological studies

**DOI:** 10.1017/rsm.2024.9

**Published:** 2025-04-01

**Authors:** Yanfei Li, Elizabeth Ghogomu, Xu Hui, E. Fenfen, Fiona Campbell, Hanan Khalil, Xiuxia Li, Marie Gaarder, Promise M. Nduku, Howard White, Liangying Hou, Nan Chen, Shenggang Xu, Ning Ma, Xiaoye Hu, Xian Liu, Vivian Welch, Kehu Yang

**Affiliations:** 1 Center for Evidence-Based Medicine, School of Basic Medical Science, Lanzhou University, Lanzhou, China; 2 Bruyère Research Institute, University of Ottawa, Ottawa, ON, Canada; 3 Campbell Collaboration, Ottawa, ON, Canada; 4 Department of Public Health and Healthcare-Associated Infection Management, Affiliated Hospital of Qinghai University, Xining, China; 5 Population Health Sciences Institute, Newcastle University, Newcastle, UK; 6 La Trobe University, School of Psychology and Public Health, Department of Public Health, Melbourne, Australia; 7 Center for Evidence-Based Social Science/Center for Health Technology Assessment, School of Public Health, Lanzhou University, Lanzhou, China; 8 International Initiative for Impact Evaluation (3ie), London, UK; 9 Pan-African Collective for Evidence (PACE), Johannesburg, South Africa; 10 Evaluation and Evidence Synthesis, Global Development Network, New Delhi, India; 11 McMaster Health Forum, McMaster University, Hamilton, Canada; 12 Research and Education Department, Shanxi Provincial Rehabilitation Hospital, Xi’an, China

**Keywords:** evidence mapping, mapping reviews, methodology, reporting, scoping reviews

## Abstract

Mapping reviews (MRs) are crucial for identifying research gaps and enhancing evidence utilization. Despite their increasing use in health and social sciences, inconsistencies persist in both their conceptualization and reporting. This study aims to clarify the conceptual framework and gather reporting items from existing guidance and methodological studies. A comprehensive search was conducted across nine databases and 11 institutional websites, including documents up to January 2024. A total of 68 documents were included, addressing 24 MR terms and 55 definitions, with 39 documents discussing distinctions and overlaps among these terms. From the documents included, 28 reporting items were identified, covering all the steps of the process. Seven documents mentioned reporting on the title, four on the abstract, and 14 on the background. Ten methods-related items appeared in 56 documents, with the median number of documents supporting each item being 34 (interquartile range [IQR]: 27, 39). Four results-related items were mentioned in 18 documents (median: 14.5, IQR: 11.5, 16), and four discussion-related items appeared in 25 documents (median: 5.5, IQR: 3, 13). There was very little guidance about reporting conclusions, acknowledgments, author contributions, declarations of interest, and funding sources. This study proposes a draft 28-item reporting checklist for MRs and has identified terminologies and concepts used to describe MRs. These findings will first be used to inform a Delphi consensus process to develop reporting guidelines for MRs. Additionally, the checklist and definitions could be used to guide researchers in reporting high-quality MRs.

## Highlights

### What is already known

Mapping reviews are systematic methods designed to identify research status and gaps to reduce resource wastage.

Their use is increasing, especially in health and social sciences, but inconsistencies in concepts and reporting standards exist among different organizations and authors.

### What is new

This study systematically analyzed existing guidance and methodological studies to elucidate and propose a consistent framework for terminologies, definitions, categorizations, timelines, and comparisons with other review methods.

It identified 28 reporting items for mapping reviews, providing a comprehensive set of evidence-based reporting recommendations.

### Potential impact for *Research Synthesis Methods* readers

The proposed framework and reporting items aim to bridge existing gaps and provide essential methodological and reporting resources for primary authors, reviewers, journal editors, and other stakeholders.

These recommendations will serve as a foundation for future methodological researchers, guideline developers, and practical researchers, promoting complete and transparent reporting in mapping reviews.

## 1 Introduction

In response to the crucial challenge of optimizing the allocation of available resources to areas with the highest potential impact and minimizing research waste, researchers and policymakers urgently require a systematic and efficient approach to identify research gaps, needs, and priorities, as well as to enhance access to high-quality evidence for decision-makers.^1^ ^–^ ^3^ Mapping reviews (MRs) represent a method of evidence synthesis that systematically collects, assesses, and synthesizes existing evidence to clarify the current state and gaps in research, thereby fostering further research and decision-making.^4^ Introduced by the Yale Prevention Research Center in 2000 as “evidence mapping,”^5^ several organizations including the Evidence for Policy and Practice Information and Coordinating Centre (EPPI-Center), the Global Evidence Mapping Initiative (GEMI) in Australia, the Social Care Institute for Excellence (SCIE), the Collaboration for Environmental Evidence (CEE), the International Initiative for Impact Evaluation (3ie), and the Campbell Collaboration have since developed specific evidence products based on this method.^6^ ^,^ ^7^ These products systematically organize and present current evidence and gaps within specific fields, providing valuable information for future research initiatives. Additionally, numerous institutions such as the World Health Organization (WHO), the United Nations International Children’s Emergency Fund (UNICEF), the U.S. Department of Veterans Affairs, and the UK Department for International Development (DFID) have employed the MR approach.^8^ ^–^ ^11^

Research findings have indicated a significant increase in the application of MRs since 2017, with these maps becoming increasingly prevalent in various fields such as medicine, environmental science, international development, and other health and social sciences.^7^ ^,^ ^12^ ^–^ ^15^ However, the absence of universally accepted reporting standards and methodological guidance has led to varied terminologies and reporting formats across different organizations and research fields, despite their shared objectives.^6^ This lack of consistency in core methodological concepts and reporting has substantially impeded the development of high-quality MRs and curtailed their potential to reduce research waste. In recent years, several guidance documents aimed at standardizing this method have been published. Noteworthy among them are the guidance by Howard White et al. for producing a Campbell evidence and gap map in social sciences,^16^ the ROSES (RepOrting standards for Systematic maps) by Neal R. Haddaway et al. for environmental sciences,^17^ and the guidance for conducting systematic mapping studies in software engineering by Kai Petersen et al.^18^ Furthermore, a methodological study by Hanan Khalil et al. in 2023 has underscored the existing methodological inconsistencies and confusion in the field.^7^ Developing a widely accepted conceptual framework and reporting recommendations that encapsulate the unique functionalities of MRs, based on existing guidance and methodological studies from various organizations and fields, represents a critical direction for future research.

This study follows the established methods for a scoping review as defined by the Joanna Briggs Institute (JBI).^19^ Its aims are to elucidate and propose a consistent framework for key concepts and collect appropriate reporting items based on existing guidance and methodological studies related to MRs.

## 2 Methods

This scoping review was conducted in accordance with the PRISMA extension for Scoping Reviews reporting guideline.^20^ The protocol for this study has been published elsewhere.^21^

### 2.1 Literature search

In the initial stage, nine electronic databases (Medline, Embase, the Cochrane Library, the Campbell Library, Web of Science Core Collection, China National Knowledge Infrastructure, VIP Chinese Science and Technique Journals Database, the Chinese Biomedical Database, Wan Fang Data) and 11 institution websites (Supplement Table S1) were searched from inception to January 2024 to ascertain the best available evidence. Following this, backward citation searching was performed for references cited in both primary studies and reviews related to the topic identified during the initial search. Furthermore, we consulted content experts in the field, particularly those on the advisory board, to further augment our resources. Simultaneously, the search strategy was determined based on the search terms, with no restrictions on date, language, or country/region. The major search terms and strategies (see Supplement Table S1) included the following: “evidence map*” or “gap map*” or “evidence gap*” or “systematic map*” or “evaluation map*” or “descriptive map*” or Megamap or Mega-map or “map of map*” or “mapping evidence” or “mapping review*.”

### 2.2 Inclusion and exclusion criteria

According to JBI guidance, the inclusion criteria were formulated using the “PCC” framework, which encompasses Population, Concept, and Context.^7^ ^,^ ^22^ In this study, population and context were deliberately omitted, implying an absence of specific restrictions, while the Concept included any guidance documents and methodological studies pertinent to MRs.

While formal guidance documents typically undergo a structured development process, similar to approaches adopted by other study,^23^ this project did not confine the review scope exclusively to papers that delineate a formal guidance development process. Instead, we aimed to encompass a broad range of perspectives by including all guidance documents, whether published in journals or not, that offer advice and specific recommendations on key concepts, steps, principles, and strategies of MRs.^16^ ^,^ ^24^ ^,^ ^25^

We included any methodological study that describes or analyzes methods in published or unpublished MRs.^7^ ^,^ ^26^ Methodological studies employ systematic methods to analyze literature gathered through systematic search techniques, often using a research report as the unit of analysis.^27^ An example of an eligible methodological study is a recent scoping review of 335 MRs by Khalil et al.^7^

We excluded duplicate literature and individual MRs.

### 2.3 Study selection and data extraction

The entire process of screening and data extraction was performed independently by two reviewers. When discrepancies arose between the reviewers, a third reviewer was consulted to resolve the differences. Before the formal screening, we conducted training and a pilot study on approximately 10% of the search results to ensure that both reviewers achieved a minimum 90% agreement level. We utilized Covidence (www.covidence.org) for literature screening. Initially, duplicate studies were identified and removed. Subsequently, two reviewers evaluated the titles and abstracts of the selected studies. Studies were removed from further review if both reviewers agreed to exclude them. If at least one reviewer included a study, or if the title and abstract provided insufficient information for a decision, the full article was obtained for further review.

A standardized data extraction form was developed specifically for extracting the desired information, utilizing Excel 2023 (Microsoft Corporation, Redmond, WA, USA). Following a pilot test of the data extraction form, teams of two reviewers independently extracted data, including descriptive information such as publication author, year, title, and country; research field, including health, social sciences: aging, business and management, children and young persons’ well-being, climate solutions, crime and justice, disability, education, international development, knowledge translation and implementation, and social welfare based on the classification standard of the Campbell Collaboration, and others^28^ ^–^ ^31^; key concepts of MRs, including employed terminology, definition, and category; and reporting characteristics including, but not limited to, the items related to titles, authors, abstracts, background, methods, results, conclusions, and funding.

### 2.4 Data analysis

We presented the results using tables and figures. Descriptive summary statistics, including frequency and median (interquartile range [IQR]), were calculated for each specified potential reporting item. Additionally, we adopted a narrative approach in cases where quantitative synthesis was not feasible. Our reporting adhered to the PRISMA for scoping reviews (PRISMA-ScR) guidelines.^20^

### 2.5 Role of the funding source

The sponsors were not involved in research design, data collection, data analysis, and report writing. The corresponding author is responsible for all aspects of the study to ensure the proper investigation and resolution of the research for problems related to accuracy or completeness. The final version was approved by all authors.

## 3 Results

### 3.1 Literature search

**Figure 1:**
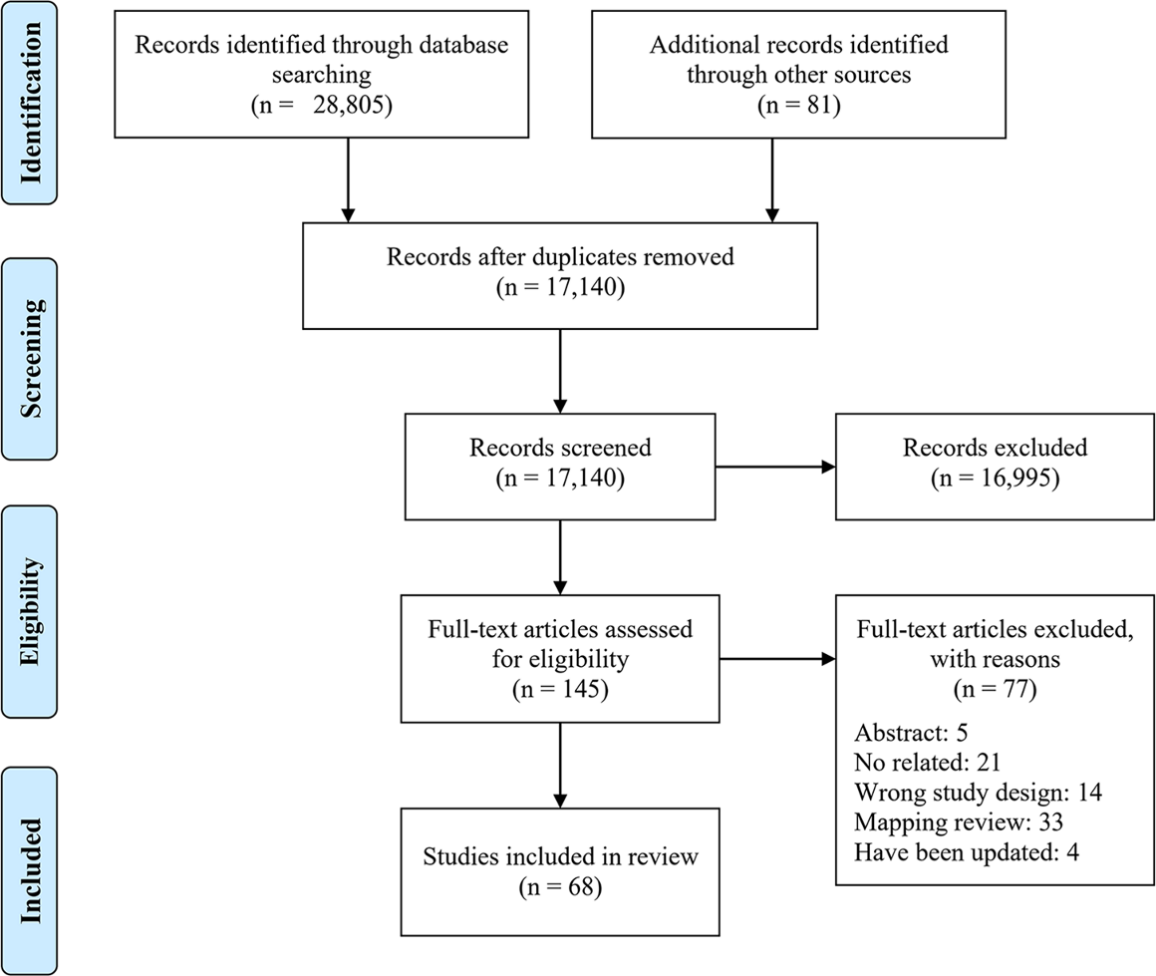
Flowchart of the literature screening process and results.

Figure 1 presents a flowchart depicting the literature selection process. Initially, 28,886 relevant records were identified. Of these, 11,746 records were removed due to duplication. The titles and abstracts of the remaining 17,140 studies were then screened, resulting in 16,995 being excluded as they did not meet the inclusion criteria. The full texts of the remaining 145 articles were further assessed for eligibility, leading to the exclusion of an additional 77 studies, detailed in Supplement Table S2. Ultimately, 68 documents met our eligibility criteria and were included in the scoping review.

### 3.2 Study characteristics

As illustrated in Table 1, a total of 68 documents were included in the scoping review, comprising 59 guidance and 9 methodological studies, predominantly journal articles (56, 82.35%). The UK contributed the most documents (24, 35.29%), followed by the USA (13, 19.12%), with Australia and China each contributing five documents (7.35%). There has been a rapid increase in the number of relevant documents over time, particularly from 2018 to 2023, during which more than 30 documents were identified. The majority of documents focused on health science (13, 19.12%) and climate solutions/environmental sciences (12, 17.65%), with 24 documents (35.29%) not specifying a research field.

**Table 1:** Characteristics of included documents

Category	*N* (%)
Document publisher	
Academic journal	56 (82.35%)
Nongovernmental organization	6 (8.82%)
Government	4 (5.88%)
University/research organization	2 (2.94%)
Document type	
Guidance document	59 (86.76%)
Methodological study	9 (13.24%)
Publication year	
2003–2007	2 (2.94%)
2008–2012	9 (13.24%)
2013–2017	18 (26.47%)
2018–2022	35 (51.47%)
2023	4 (5.88%)
Country	
UK	24 (35.29%)
USA	13 (19.12%)
Australia	5 (7.35%)
China	5 (7.35%)
German	4 (5.88%)
Others	17 (25%)
Research field	
Health science	13 (19.12%)
Climate solutions/environmental sciences	12 (17.65%)
Others-software engineering	5 (7.35%)
Social welfare	4 (5.88%)
Crime and justice	1 (1.47%)
Education	1 (1.47%)
International development	1 (1.47%)
NR	24 (35.29%)
Others	7 (10.29%)

*Note*: NR, not reported indicates that there are no restrictions on the research field for entries in this table.

### 3.3 Frequency and field distribution of terminology

As indicated in Table 2 and the Supplement Table S3, the included studies mentioned 24 research terms related to MRs. The term “evidence map” was the most frequently used, appearing in 23 documents and accounting for 33.82% of all included documents. This was followed by “evidence mapping” (19, 27.94%), “systematic map” (17, 25%), “evidence and gap map” (15, 22.06%), and “mapping review” (7, 10.29%). Regarding the timeline of term usage, “evidence mapping” and “evidence map” appeared earliest,^5^ while “mapping review/systematic map” was categorized as one of the 14 review types in a study published by Maria J. Grant et al. in 2009.^4^ The terms “mapping review” and “evidence gap map” were adopted in guidance and methodological studies most recently published in 2023.

**Table 2:** Terminologies used in included documents and corresponding research fields

No.	Terms	*N*	Research field (*n*)
1	Evidence map	23	Health science (11), NR (8), social welfare (1), others-public health (1), others-public sector (1), international development (1)
2	Evidence mapping	19	Health science (10), NR (6), others-public health (2), climate solutions (1)
3	Systematic map	17	Climate solutions (8), social welfare (3), NR (3), health science (1), others-public sector (1), international development (1)
4	Evidence and gap map	15	NR (7), others-social science (3), climate solutions (1), others-public health (1), others-social policy (1), education (1), international development (1)
5	Mapping review	7	NR (6), health science (1)
6	Systematic mapping	5	Climate solutions (3), social welfare (2)
7	Mapping study	4	Others-software engineering (1), NR (3)
8	Systematic mapping study	4	Others-software engineering (4)
9	Map of maps	3	NR (1), others-social science (1), others-public health (1)
10	Systematic evidence map	3	Climate solutions (3)
11	Systematic evidence mapping	3	Climate solutions (3)
12	Focused mapping review and synthesis	2	Others-social science (1), NR (1)
13	Mega-map	2	Others-social science (1), others-public health (1)
14	Systematic mapping review	2	NR (2)
15	3ie map	1	Others-public sector (1)
16	Evidence review map	1	Climate solutions (1)
17	Evidence review mapping	1	Climate solutions (1)
18	Evidence/gaps mapping reviews	1	NR (1)
19	Evidence-based policing matrix	1	Crime and Justice (1)
20	Gap map	1	NR (1)
21	Glaserian systematic mapping study	1	Others-software engineering (1)
22	Mapping research	1	NR (1)
23	Rapid evidence mapping	1	Health science (1)
24	Systematic literature mapping	1	Social welfare (1)

*Note*: NR, not reported indicates that there are no restrictions on the research field for entries in this table.

Regarding the distribution of research fields (Table 2), documents using the terms “evidence map” and “evidence mapping” primarily focus on health sciences (11, 47.83%; and 10, 52.63%, respectively) and often do not specify a research field (8, 34.78%; and 6, 31.58%, respectively). Documents utilizing “evidence and gap map” typically address social sciences such as education and climate solutions (7, 46.67%). Those labeled as “systematic map/mapping,” “systematic evidence map/mapping,” and “evidence review map/mapping” predominantly target climate solutions in environmental sciences (8, 47.06%; 3, 60%; 3, 100%; and 1, 100%, respectively). “Mapping review” documents are generally not field-specific (6, 85.71%). Documents associated with “systematic mapping study” specifically concentrate on the software engineering field (4, 100%).

### 3.4 Definition components and objectives

**Figure 2:**
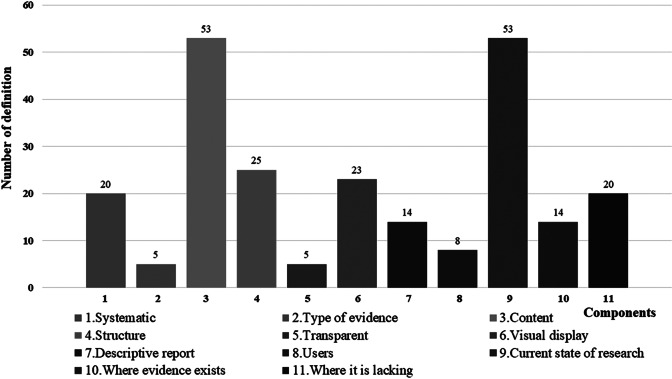
Analysis of 55 definitions across 11 components.

As shown in Figure 2 and Supplement Table S4, a total of 55 definitions describing various terms or methods were analyzed, revealing that “systematic” was mentioned in 20 definitions (36.36%), “type of evidence” in 5 (9.09%), “content” in 53 (96.36%), “structure” in 25 (45.45%), “transparent” in 5 (9.09%), “visual display” in 23 (41.82%), “descriptive report” in 14 (25.45%), and “users” in 8 (14.55%). Furthermore, 53 definitions (96.36%) across various terms mentioned the objective of using relevant methods/tools to determine the current state of research, of which 14 (25.45%) further mentioned where evidence was present, and 20 mentioned where it was lacking (36.36%). Key distinctions in definitions were notably present in “type of evidence” and “visual display.” Specifically, “type of evidence” focused solely on evidence for interventional management in three definitions (37.5%) for “evidence and gap map” and one (100%) for “evidence-based policing matrix.” Meanwhile, “visual display” involved the creation of an interactive database in one definition each for “evidence and gap map” (12.5%) and “systematic map” (20%).

### 3.5 Terminology variations

Thirty-nine out of 68 documents discuss variations in terminology (Supplement Table S3). Seventeen (43.59%) documents examine the relationship between “evidence mapping” and “evidence map,” with nine of them (52.94%) reporting that “mapping” is a process or methodology, and a “map” is a multidimensional presentation of included studies, seven documents (41.18%) mention that these terms are used together without specifying differences, and one document (5.88%) considers an “evidence map” as a subgroup of “evidence mapping.” Seven documents (17.95%) explore the relationship between “systematic mapping” and “systematic map,” with six (85.71%) indicating that these are used interchangeably. Five documents (12.82%) investigate the usage of terms like “evidence map,” “mapping review,” “systematic map,” “mapping study,” and “mapping research,” noting a general lack of specified differences in their application. Additionally, one study (2.56%) introduces “evidence review mapping/map” as a new method focused solely on the review landscape for specific environmental topics or questions, and another describes “focused mapping review and synthesis” (FMRS) as a new review form.

Five documents (12.82%) analyze the particularity of evidence and gap map (EGM), noting that, unlike other maps, EGMs typically only include systematic reviews (SRs) and impact evaluations (IEs), with the “DPME policy relevant Evidence map” potentially including other types of research as well. EGMs are particularly noted for their intervention–outcome framework visualization and provision of user-friendly summaries. Similarly, the “evidence-based policing matrix” is described as including only IEs. Two documents (5.13%) mention the relationship between EGM, “mega-map,” and “map of maps,” stating that “mega-map” includes only SRs and other maps, while “map of maps” includes only other maps. One document (2.56%) highlights the difference between “mapping review” and EGM, stating that EGMs can either accompany an MR as a visual representation or stand independently.

Based on the analysis, as a systematic method, “mapping review” updates and replaces earlier terms such as “evidence mapping” and others including “evidence/gaps mapping reviews,” “mapping research/study.”^4^ ^,^ ^32^ ^–^ ^34^ It encompasses terms used in specific fields like “systematic map” for environmental science,^17^ “systematic mapping study” for software engineering.^18^ Additionally, it includes newly recognized independent methodologies like FMRS,^35^ and “evidence review mapping.”^36^ The term “evidence map” is consistently used within MRs in various forms such as tables, charts, or databases. An EGM is a special interactive form of an evidence map that can be used within MRs, other research methodologies, or as a standalone tool.^6^ ^,^ ^7^ ^,^ ^25^ ^,^ ^37^ ^,^ ^38^ The systematic definition and objective of MRs are as follows: MRs are a method of evidence synthesis that systematically collects, assesses, and synthesizes existing evidence to clarify the current state and gaps in research, thereby fostering decision-making and further research. It aims to identify areas with adequate evidence to support decision-making and highlight areas lacking evidence to guide primary research and evidence synthesis.

### 3.6 Timeline and comparison with other methods

As shown in Supplement Table S5, 13 documents reported the time frame required to complete an MR. Among them, five (38.46%) reported a required time frame of 1–6 months, another five reported a required time frame of 6–12 months, and three (23.08%) reported a required time frame of more than 12 months.

Twenty-eight documents discussed the differences between MRs and systematic reviews. Among them, 23 documents (82.14%) mentioned the research topic and objectives. Systematic reviews focus on specific research questions and synthesize studies qualitatively or quantitatively to address these questions. In contrast, MRs focus on broader research areas, aiming to identify the current distribution of evidence. MRs also have systematic searches but can be adjusted for different purposes (16, 57.14%), include all types of study designs (10, 35.71%), do not necessarily include quality assessments (16, 57.14%), and involve coding interesting characteristics instead of extracting research findings (6, 21.43%). Data analysis in MRs uses visualization to present evidence from different fields (19, 67.86%), resulting in an evidence map rather than a summary of effects (10, 35.71%). Stakeholder involvement is recommended in MRs (1, 3.57%).

Twenty-two documents discussed the differences and connections between MRs and scoping reviews. Among them, 11 documents (50.00%) noted similarities in research topics, searches, screening, and inclusion/exclusion criteria. Compared to scoping reviews, MRs typically include a standardized or consensus-based data coding process (5, 22.73%), and quality assessment is recommended (but not mandatory), while it is generally not suggested for scoping reviews (3, 13.64%). MRs often recommend early stakeholder involvement (2, 9.09%), and their final output is an evidence map rather than a summary table of study content (7, 31.82%). The aim of MRs is to discover the current distribution of evidence rather than to summarize the content of the included studies. MRs can be distinguished from scoping reviews because the subsequent outcome may involve further review work or primary research, and this outcome is not known beforehand (11, 50.00%).

### 3.7 Reporting recommendations

In the 68 included documents, 57 mentioned one or more reporting recommendations for MRs, covering 28 items related to topics such as Title, Authors, Abstract, Background, Methods, Results, Discussion, Conclusions, Acknowledgements, Contributions of authors, Declarations of interest, and Sources of support. According to our protocol,^21^ the research team (KHY, VW, EG, YFL, XH, LYH, NM, NC, FFE, XYH, SGX, and XL) summarized the different recommendations on the same topics using a predefined data extraction form and refined a new set of items that attempted to align with all the suggestions (Supplement Tables S6–9). Next, all the advisory board members (MG, HW, PN, FC, HK, and XXL) held a consensus meeting, in which the wording of 15 items was revised, and consensus was reached on each item (with materials from the meeting uploaded to OSF^21^).

**Table 3:** Twenty-eight items (including 39 recommendations) identified from guidance and methodological studies

Section	Topic	Item #	Checklist
Title	Title	1	Specify the scope and identify the report as a mapping review, evidence and gap map, or both
Authors	Authors	2	List names and affiliations of all authors
Abstract	Structured summary	3	Provide a structured summary that includes (as applicable) background, including the rationale and objective for the review*; methods, including stakeholder engagement, protocol, eligibility criteria, search, coding, critical appraisal, and data presentation and analyses (specifically mention the strategy for adequacy and priority setting); results, including study selection, characteristics, risk of bias, and mapping analyses; conclusions, including a summary of the main findings and implications of those findings
Background	Rationale	4	Describe the scope of the review and explain why conducting this review is important
	Objectives	5	Provide a structured statement of the research question(s) within a key element framework, and specify whether this review is intended for decision-making, to delineate evidence gaps and clusters for future research, or both. Utilize frameworks such as PI/ECOS (Population, Intervention/Exposure, Comparator, Outcome, and Study Design), PCC (Population, Concept, and Context), PIT (Population, Index Test, Target Condition), and others like PECO, PEO, and PO for various research questions.
Methods	Stakeholders		
	Identification and definition of stakeholders	6a	Specify the identification process of stakeholders and define the various types of stakeholders involved, such as direct users of research outputs (e.g., researchers and policy decision-makers), and those directly affected by decisions (e.g., patients in the field of medicine)
	Stakeholder engagement	6b	Provide detailed descriptions of stakeholder engagement at each stage of the review process
	Registration and protocol		
	Registration information	7a	Provide registration information for the review, or state if the review was not registered
	Reference protocol	7b	Apply the review protocol, or specify if no protocol was established
	Deviations from protocol	7c	Describe and explain any amendments made to the information provided at registration or in the protocol
	Eligibility criteria	8	Specify the inclusion and exclusion criteria for the review, defining characteristics of the study within a key element framework based on different research questions.
	Search sources	9	Present all databases, registers, websites, organizations, reference lists, and other sources searched or consulted to identify studies, along with the dates when each source was last searched or consulted. If machine learning was utilized for literature search, present the details of the sources from which the literature was drawn
	Search strategy	10	Present the full search strategies for all search sources, including any filters and limits used. If machine learning was utilized for literature search, present the details of the relevant software and its search strings
	Screening process	11	Provide the process for literature screening, including at the title/abstract and full texts levels, and clarify the methods ensuring the repeatability of this process, such as specifying the number of people involved and whether they worked independently. If machine learning was used for literature screening, specify the details of how inclusion decisions were made
	Data extraction and coding		
	Data items	12a	List all variables used for data extraction and/or further coding
	Development of coding tools	12b	Define and provide details on the analytic or consensus-based conceptual framework developed for developing coding tools
	Data collection process	13	Provide the process for data extraction from reports of included studies and coding, and clarify the methods ensuring the repeatability of this process, such as specifying the number of people involved and whether they worked independently. If machine learning was used for data extraction, specify the details of the relevant software and the logic used for extracting key fields.
	Critical appraisal	14	Specify whether the quality of included studies or reviews was assessed. If done, describe the methods used for assessment and the procedures ensuring the repeatability of the assessment process, such as specifying the number of people involved and whether they worked independently.
	Data presentation and analysis		
	Types of presentation	15a	Specify the presentation formats for the mapping results of included studies, such as charts, tables, and interactive maps
	Tools for mapping	15b	Describe the tools or software details used for generating mapping results, including any automation or artificial intelligence software
	Dimensions in Maps	15c	Specify the dimensions or coordinate matrix and filters (if possible) used for positioning within the maps. If Evidence Gap Maps (EGMs) tools are utilized, consult the PRISMA-EGM guidelines as necessary
	Strategy for adequacy and priority setting	15d	Specify the strategy for determining areas with sufficient evidence, aimed at supporting decision-making and future research priorities, including evidence gaps and clusters. If possible, specify the method for ranking these priorities.
	Data analysis methods	15e	Describe the methods used in the data analysis process, such as descriptive analysis, thematic analysis, and statistical analysis, among others
Results	Study selection		
	Flow of studies	16a	Describe the results of the search and selection process, from the number of references initially identified to the number of studies ultimately included in the review. Provide a flow diagram to illustrate this process
	Excluded studies	16b	List key excluded studies that readers might reasonably expect to find and provide justification for each exclusion
	Study characteristics	17	Describe the basic study characteristics of interest. Consider equity and provide the citations, native format of extracted data and its corresponding coding for each included study, if possible.
	Quality assessments	18	If quality was assessed, describe the quality assessments for included studies or reviews.
	Mapping analysis		
	Maps of included studies	19a	Present a map here, showing how the relevant literature is organized according to transparent, replicable key elements framework (research question), with a concise description. If possible, consider equity and include filters that allow for the generation of customized maps.
	Areas with adequate evidence	19b	Provide a structured report within a key element framework detailing areas with sufficient evidence support for decision-making based on the strategy for adequacy, if possible
	Evidence gaps and clusters	19c	Provide a structured report within a key element framework detailing areas requiring further research, including primary studies and additional evidence synthesis, based on the strategy for priority setting, if possible
Discussion	Summary of main results	20	Describe the main findings and provide a general interpretation of the results within the context of other evidence
	Limitations of the review	21	Discuss the limitations of both the review processes and the included evidence
	Implications	22	Discuss the implications for decision-making and future research in a structured manner based on key elements framework, if possible
	Plans for map updates	23	Discuss the necessity of updating the map based on current research results and trends, and provide details of the update plan, including timing and content to be updated.
Conclusions	Conclusions	24	Report the main findings of the review and summarize the implications of those findings
Acknowledgements	Acknowledgements	25	Acknowledge the contributions of individuals who are not listed as authors of the review
Contributions of authors	Contributions of authors	26	Detail the specific contributions of each author
Declarations of interest	Declarations of interest	27	Report any competing interests of the review authors
Sources of support	Sources of support	28	Describe the sources of financial or non-financial support for the review and specify the role of the funder

* The term “review” in the items includes the EGM report.

The 28 reporting items formed are shown in Table 3. Among the 68 documents included, seven documents (six guidance documents and 1 methodological study) mentioned reporting on the title of an MR. Three guidance documents mentioned the authors, and four mentioned reporting structured abstracts. Fourteen documents (12 guidance documents and 2 methodological studies) discussed the reporting of rationale and objectives in the background section. Fifty-six documents (48 guidance documents and 8 methodological studies) addressed reporting on 10 methods-related items (stakeholders, registration and protocol, eligibility criteria, search sources, search strategy, selection process, data extraction and coding, data collection process, critical appraisal, and data presentation and analysis), with the median number of documents supporting an item being 34 (27, 39). Eighteen documents (15 guidance documents and 3 methodological studies) mentioned reporting on 4 results-related items (study selection, study characteristics, risk of bias in included studies, and mapping analysis), with the median number of documents supporting an item being 14.5 (11.5, 16). Twenty-five documents (23 guidance documents and 2 methodological study) discussed 4 items related to the discussion section (summary of main results, limitations of the review, implications, and plans for map updates), with the median number of documents supporting an item being 5.5 (3, 13). Six guidance documents mentioned reporting on conclusions, two guidance documents discussed acknowledgements and contributions of authors, three guidance documents mentioned declarations of interest, and four documents (3 guidance documents and 1 methodological study) discussed reporting sources of support (Supplement Tables S6–9).

Several specific reporting items were noteworthy. In the methods-related items, 22 documents (21 guidance documents and one methodological study) mention and define stakeholders, with two guidance documents (9.52%) specifically detailing the identification process of stakeholders. Additionally, 31 documents (27 guidance documents and four methodological studies) elaborate on the roles of stakeholders during various stages of development; 35 documents (32 guidance documents and three methodological studies) discuss the reporting of eligibility criteria for MRs, of which 24 (68.57%) also reporting a key elements framework for developing these criteria, including frameworks like PI/ECOS (population, intervention/exposure, comparator, outcome, and study design), PCC (population, concept, and context), PIT (population, index test, and target condition), PECO, PEO, and PO; 41 documents (36 guidance documents and five methodological studies) mention the search sources for MRs, with 31 (75.61%) explicitly reporting the use of systematic and comprehensive search methods; 39 documents (35 guidance documents and four methodological studies) discuss search strategies for MRs based on eligibility criteria or research questions, with 17 (44.74%) specifying validated search strategies for different resources. The primary reported limitations in research relate to study design, with five documents include multiple types of study designs (various primary studies and systematic reviews), two focusing only on reviews and guidelines, and one exclusively on SRs.

Furthermore, 37 documents (33 guidance documents and 4 methodological studies) mention data extraction and coding, with 26 (70.27%) mentioning the development of coding tools. Among these, 17 (65.38%) recommend that this process should utilize an analytic or consensus-based conceptual framework; 27 documents (25 guidance documents and 2 methodological studies) discuss the critical appraisal in MRs, with 13 (48.15%) recommend conducting critical appraisal (i.e., assessing the quality of individual studies) for all included studies. Seven (25.93%) suggest that quality assessment should be confined to SRs, not original studies, and eight (29.63%) view quality assessment as optional; 44 documents (37 guidance documents and 7 methodological studies) mention data presentation and analysis, all referring to types of presentation. Twenty-six documents mention presenting evidence in charts, 19 in tables, and 24 in interactive maps/databases, including tools like EGM; 15 documents mention the strategy for adequacy and priority setting, explaining how MRs utilize the evidence base to identify areas with adequate evidence to support decision-making, as well as to discover existing evidence gaps and clusters to set priorities for future research.

## 4 Discussion

In this scoping review, we examined guidance and methodological studies related to MRs. We uncovered substantial inconsistencies in core concepts and reporting practices. To address these inconsistencies, our research proposes a key conceptual framework derived from existing guidance and methodological studies. This framework includes standardized terminology and categorization, clear definitions, precise objectives, and comprehensive reporting recommendations.

We identified 24 relevant terms and 55 definitions for these terms (any descriptive statements). Our systematic analysis examined the occurrence of these terms over time, their focus fields, the 11 definition components identified by Ashrita Saran et al.,^6^ and distinctions made between different types of MRs in the included documents. We found that primary distinctions in existing terms relate more to the research field than to the methodology itself. Terms such as “mapping review,” “evidence mapping,” “evidence/gaps mapping reviews,” and “mapping research/study” were found to describe the same methodological approach, a finding that is partially or fully supported by several included guidance documents^32^ ^,^ ^34^ ^,^ ^39^ ^–^ ^43^ and a methodological study.^33^ Notably, while the term “evidence map” is widely used and prevalent, the majority of relevant guidance documents still regard it as a tool rather than a methodological approach.^5^ ^,^ ^44^ ^–^ ^49^ This tool consistently appears in MRs, invariably taking the form of charts, tables, or interactive databases, and is also observed in scoping reviews and network meta-analyses.^6^ ^,^ ^20^ ^,^ ^26^ ^,^ ^37^ ^,^ ^50^ The EGM, as a special type of map,^37^ ^,^ ^38^ ^,^ ^51^ ^–^ ^53^ systematically presents evidence through an interactive database and can be utilized within MRs, other research methodologies, or independently.^7^ Importantly, MRs differ from other review types in that they are not focused on resolving clinical issues, determining the effectiveness of interventions (like SRs^54^), or summarizing evidence (what the evidence says; like scoping reviews^19^). Instead, MRs focus on identifying where evidence exists and where there are evidence gaps.^25^ ^,^ ^53^ This focus is crucial for researchers and decision-makers to understand the landscape of available research and to prioritize future studies.^5^ ^–^ ^7^ ^,^ ^37^

Based on the documents included, we formed 28 recommended items, each supported or mentioned by more than one document. We identified three checklists related to MRs,^16^ ^,^ ^17^ ^,^ ^20^ two of which^16^ ^,^ ^17^ were specifically designed for MRs. Neal R. Haddaway et al.^17^ developed reporting standards for environmental science MRs (systematic maps), while Howard white et al.^16^ ^,^ ^55^ created a reporting checklist for social science MRs (Campbell EGMs). Our preliminary set of 28 recommended items is derived from these checklists, enhanced and refined with insights from other guidance documents and methodological studies included. For example, stakeholder engagement, a critical feature of MRs, is detailed in our list under the identification and definition of stakeholders, and stakeholder engagement at each stage of the review process based on included documents.^35^ ^,^ ^36^ ^,^ ^56^ ^–^ ^60^ Additionally, we have refined and developed new items that align with the distinctive features of MRs. These enhancements ensure suitability across various disciplines and fields, improve methodological effectiveness, incorporate the use of machine learning,^61^ ^–^ ^65^ and consider equity.^7^ Specific examples of these new items include those related to objectives, strategy for adequacy and priority setting, and mapping analysis.

## 5 Strengths and limitations

We employed the JBI scoping review methodology^19^ to systematically gather and analyze guidance documents and methodological studies from diverse sources—including academic journals, nongovernmental organizations, governments, and university/research organizations—across various research fields. This scoping review meticulously defined the core concepts of MRs, effectively addressing and mitigating the research gaps caused by the existing confusion over terminologies and definitions within this methodology. The results will provide a crucial foundation for future methodological research and practice in this field. Additionally, our multidisciplinary team, each with at least 3 years of relevant field experience, developed a reporting item checklist applicable across various fields, based on the documents included. This checklist will serve as a critical reference for the reporting of MRs. However, there are some limitations to our study. First, as this method is rapidly evolving, there are significant discrepancies in opinions among different organizations and authors. In this context, our conclusions are based on the majority’s research rather than the consensus of all involved. Second, our items were solely dependent on the relevant documents and the opinions of the project’s advisory committee. These items have not undergone a formal Delphi survey, but they will serve as an essential reference for future methodological research, especially in the development of reporting guidelines, and MR practices.

## 6 Conclusion

Currently, there is a significant divergence among different organizations and authors regarding the key concepts of MRs and the standards for complete and transparent reporting. This lack of unified guidance affects primary authors, reviewers, journal editors, and other stakeholders. Based on a systematic analysis of all relevant terms, definitions, timelines, comparisons with other methods, and reporting characteristics from the 68 included guidance and methodological studies, we have proposed terminologies, definitions, categorizations, and reporting recommendations for MRs. These proposals aim to bridge existing gaps and provide essential methodological and reporting resources for this field. This comprehensive set of evidence-based reporting items will also serve as a foundation for future methodological researchers, guideline developers, and practical researchers.

## Supporting information

Li et al. supplementary material Li et al. supplementary material

## Data Availability

The data analyzed in this study is contained in the manuscript and its supporting information.

## Supplementary material

To view supplementary material for this article, please visit http://doi.org/10.1017/rsm.2024.9.
