# Supplementary material for: Key concepts and reporting recommendations for mapping reviews: A scoping review of 68 guidance and methodological studies
Source: Res Synth Methods. 2025 Apr 1;16(1):157–74. doi: 10.1017/rsm.2024.9 (PMC12631146; doi:10.1017/rsm.2024.9)
Supplement: Li et al. supplementary material [file S1759287924000097sup001.docx]

**Key concepts and reporting recommendations for mapping reviews: a scoping review of 68 guidance and methodological studies**

Yanfei Li^1,2^, Elizabeth Ghogomu^2,3^, Xu Hui^1^, Fenfen E^4^, Fiona Campbell^5^, Hanan Khalil^6^, Xiuxia Li^7^, Marie Gaarder^8^, Promise M. Nduku^9^, Howard White^1,10^, Liangying Hou^1,11^, Nan Chen^12^, Shenggang Xu^7^, Ning Ma^1^, Xiaoye Hu^7^, Xian Liu^7^, Vivian Welch^2,3^ and Kehu Yang^1^

^1^Center for Evidence-Based Medicine, School of Basic Medical Science, Lanzhou University, Lanzhou, China

^2^Bruyère Research Institute, University of Ottawa, Ottawa, Canada

^3^Campbell Collaboration, Ottawa, Canada

^4^Department of Public Health and Healthcare-associated Infection Management, Affiliated Hospital of Qinghai University, Xining, China

^5^Population Health Sciences Institute, Newcastle University, Newcastle, UK

^6^La Trobe University, School of Psychology and Public Health, Department of Public Health, Melbourne, Australia

^7^Center for Evidence-Based Social Science/Center for Health Technology Assessment, School of Public Health, Lanzhou University, Lanzhou, China

^8^International Initiative for Impact Evaluation (3ie), London, UK

^9^Pan-African Collective for Evidence (PACE), Johannesburg, South Africa

^10^Evaluation and Evidence Synthesis, Global Development Network, New Delhi, India

^11^McMaster Health Forum, McMaster University, Hamilton, Canada

^12^Research and education department, Shanxi Provincial Rehabilitation Hospital, Xi’an, China

**Contents**

[Supplement Table 1. Search strategies 3](#_Toc173325917)

[Supplement Table 2. Excluded studies 5](#_Toc173325918)

[Supplement Table 3. Terminology, focus research fields, and terminology variations in 68 included documents 9](#_Toc173325919)

[Supplement Table 4. Components and objectives of 55 definitions 17](#_Toc173325920)

[Supplement Table 5. Timeline for developing mapping reviews and comparison with other methods 35](#_Toc173325921)

[Supplement Table 6. Potential reporting characteristics for title, author, abstract, and background of mapping reviews 39](#_Toc173325922)

[Supplement Table 7. Potential reporting characteristics for methods of mapping reviews 48](#_Toc173325923)

[Supplement Table 8. Potential reporting characteristics for results of mapping reviews 54](#_Toc173325924)

[Supplement Table 9. Potential reporting characteristics for discussion, conclusions, and other items of mapping reviews 68](#_Toc173325925)

**Supplement Table 1. Search strategies**

| **Databases [Platform]** Searches run January 2024 | **Results** |
| --- | --- |
| MEDLINE (Ovid) | 9307 |
| Embase (Ovid) | 11259 |
| Web of Science | 6998 |
| The Campbell Library | 19 |
| The Cochrane Library (Ovid)) | 604 |
| China National Knowledge Infrastructure | 427 |
| VIP Chinese Science and Technique Journals Database | 104 |
| The Chinese Biomedical Database | 41 |
| Wanfang Data | 46 |
| Other resources | 81 |
| TOTAL | **28886** |
| Duplicate | **11746** |

| **Database** | **Search strategy** | **Results** |
| --- | --- | --- |
| MEDLINE (Ovid) | #1 (evidence adj2 (map* or gap*)).ti,ab,kf. | 5545 |
|  | #2 (gap adj2 map*).ti,ab,kf. | 349 |
|  | #3 ((Systematic or Evaluation or Descriptive) adj2 map*).ti,ab,kf. | 1666 |
|  | #4 (Megamap or Mega-map or "map of map*").ti,ab,kf. | 1871 |
|  | #5 ("mapping evidence" or "mapping review*").ti,ab,kf. | 518 |
|  | #6 1 or 2 or 3 or 4 or 5 | **9307** |
| Embase (Ovid) | #1 (evidence adj2 (map* or gap*)).ti,ab,kf. | 6707 |
|  | #2 (gap adj2 map*).ti,ab,kf. | 327 |
|  | #3 ((Systematic or Evaluation or Descriptive) adj2 map*).ti,ab,kf. | 1972 |
|  | #4 (Megamap or Mega-map or "map of map*").ti,ab,kf. | 2316 |
|  | #5 ("mapping evidence" or "mapping review*").ti,ab,kf. | 533 |
|  | #6 1 or 2 or 3 or 4 or 5 | **11259** |
| Web of Science | "evidence map*" or "gap map*" or "evidence gap*" or "Systematic map*" or "Evaluation map*" or "Descriptive map*" or Megamap or Mega-map or "map of map*" or "mapping evidence" or "mapping review*" (Topic) | **6998** |
| The Campbell Library | Applied Filters: EVIDENCE AND GAP MAP | **19** |
| The Cochrane Library (Ovid)) | #1 (evidence adj2 (map* or gap*)).ti,ab,kf. | 451 |
|  | #2 (gap adj2 map*).ti,ab,kf. | 5 |
|  | #3 ((Systematic or Evaluation or Descriptive) adj2 map*).ti,ab,kf. | 65 |
|  | #4 (Megamap or Mega-map or "map of map*").ti,ab,kf. | 84 |
|  | #5 ("mapping evidence" or "mapping review*").ti,ab,kf. | 5 |
|  | #6 1 or 2 or 3 or 4 or 5 | **604** |
| China National Knowledge Infrastructure | 主题：证据图谱 + 证据图 + 证据差距图 + 差距图 + 证据地图 + 差距地图 + 证据差距地图 | **427** |
| VIP Chinese Science and Technique Journals Database | 题名或关键词=证据图谱 OR 证据图 OR 证据差距图 OR 差距图 OR 证据地图 OR 差距地图 OR 证据差距地图 | **104** |
| The Chinese Biomedical Database | ( "证据图谱"[常用字段:智能] OR "证据图"[常用字段:智能] OR "证据差距图"[常用字段:智能] OR "差距图"[常用字段:智能] OR "证据地图"[常用字段:智能] OR "差距地图"[常用字段:智能] OR "证据差距地图"[常用字段:智能]) | **41** |
| Wanfang Data | "证据图谱" OR "证据图" OR "证据差距图" OR "差距图" OR "证据地图" OR "差距地图" OR "证据差距地图" | **46** |
| Other resources  1. 3ie (http://www.3ieimpact.org/en/evidence/)  2. Evidence for Policy and Practice Information and Co-ordinating Centre (EPPI-Centre) (http://eppi.ioe.ac.uk/cms/Default.aspx?tabid=56)  3. Collaboration for Environmental Evidence (CEE)(http://www.environmentalevidence.org/)  4. The UK Department for International Development (DFID) (https://www.gov.uk/dfid-research-outputs)  5. Department of Planning, Monitoring and Evaluation (DPME), South Africa (http://www.dpme.gov.za/Pages/default.aspx)  6. Oxfam Humanitarian Evidence Programme (http://policy-practice.oxfam.org.uk/ourwork/humanitarian/humanitarian-evidenceprogramme)  7. Swedish Agency For Health Technology Assessment and Assessment of Social Services (http://www.sbu.se/en/)  8. UNICEF (https://www.unicef.org)  9. USAID (http://eccnetwork.net/resources/evidence-gapmaps/)  10. International Rescue Committee (www.rescue.org)  11. Evidence based Synthesis Programme (Department of Veteran affairs) (<https://www.hsrd.research.va.gov/publications/esp/reports.cfm>)  Consultant expert  Backward citation searching for references cited | | **81** |

**Supplement Table 2. Excluded studies**

| **Number** | **Title** | **Reason for Exclude** |
| --- | --- | --- |
| 1 | Systematic mapping - a new development in the evidence base for social care [abstract] | Abstract |
| 2 | Methods for identifying and displaying research gaps | Abstract |
| 3 | EVIDENCE GAP MAP (EGM)S: A POLICY MAKERS TOOL FOR NAVIGATING THE EVIDENCE LANDSCAPE. A CASE STUDY OF EMPLOYMENT AND HEALTH FROM THE SYSTEMS SCIENCE IN PUBLIC HEALTH AND HEALTH ECONOMICS RESEARCH (SIPHER) PROJECT | Abstract |
| 4 | Invited Perspective: The Promise of Fit-for-Purpose Systematic Evidence Maps for Supporting Regulatory Health Assessment | Abstract |
| 5 | Differentiating between mapping reviews and evidence gap maps | Abstract |
| 6 | Evidence-based mapping of design heterogeneity prior to meta-analysis: a systematic review and evidence synthesis | No related |
| 7 | 网状Meta分析相关术语和定义的研究 | No related |
| 8 | From a systematic review to addressing evidence gaps | No related |
| 9 | On the need to update systematic literature reviews | No related |
| 10 | A scoping review describes methods used to identify, prioritize and display gaps in health research | No related |
| 11 | Key stakeholders' perspectives and experiences with defining, identifying and displaying gaps in health research: a qualitative study | No related |
| 12 | Syntheses Synthesized: A Look Back at Grant and Booth's Review Typology | No related |
| 13 | How we promote rigour in systematic reviews and evidence maps at Environment International | No related |
| 14 | 证据生态系统中证据合成与转化研究方法进展与挑战 | No related |
| 15 | From agenda to action: JBI Evidence Syntheses and the United Nations sustainable development goals | No related |
| 16 | A rapid priority setting exercise combining existing, emergent evidence with stakeholder knowledge identified broad topic uncertainties | No related |
| 17 | Analytic transparency is key for reproducibility of agricultural research | No related |
| 18 | The need for multivocal literature reviews in software engineering: complementing systematic literature reviews with grey literature | No related |
| 19 | Development of literature search strategies for evidence syntheses: pros and cons of incorporating text mining tools and objective approaches | No related |
| 20 | Scientific mapping to identify competencies required by industry 4.0 | No related |
| 21 | The educational value of mapping studies of software engineering literature | No related |
| 22 | Evidence-based software engineering | No related |
| 23 | Practical use of medical terminology in curriculum mapping | No related |
| 24 | The politics of evidence and methodology: lessons from the EPPI-Centre | No related |
| 25 | Describing and analysing studies | No related |
| 26 | Visual methodology: Previously, now and in the future | No related |
| 27 | A Model-Based Approach to Systematic Review of Research Literature | Wrong study design |
| 28 | The role of scoping reviews in reducing research waste | Wrong study design |
| 29 | Evidence-Based Intervention (EBI) Mapping: a systematic approach to understanding the components and logic of EBIs | Wrong study design |
| 30 | Scoping studies: towards a methodological framework | Wrong study design |
| 31 | Procedures for performing systematic reviews | Wrong study design |
| 32 | Research priorities for public mental health in Europe: recommendations of the ROAMER project | Wrong study design |
| 33 | Lessons from applying the systematic literature review process within the software engineering domain | Wrong study design |
| 34 | Science mapping: A systematic review of the literature | Wrong study design |
| 35 | Guidelines for including grey literature and conducting multivocal literature reviews in software engineering | Wrong study design |
| 36 | Evidence-Based Software Engineering and Systematic Reviews | Wrong study design |
| 37 | What Works Clearinghouse: Procedures handbook (version 4.1) | Wrong study design |
| 38 | Guidelines for performing Systematic Literature Reviews in Software Engineering | Wrong study design |
| 39 | Sport event legacy: A systematic quantitative review of literature | Wrong study design |
| 40 | An introduction to systematic reviews. An introduction to systematic reviews | Wrong study design |
| 41 | A Systematic Map and Synthesis Review of the Effectiveness of Personal Development Planning for Improving Student Learning | Mapping review |
| 42 | Effective stakeholder participation in setting research priorities using a Global Evidence Mapping approach | Mapping review |
| 43 | What can qualitative research do for randomised controlled trials? A systematic mapping review | Mapping review |
| 44 | A Systematic Mapping Study on Requirements Scoping | Mapping review |
| 45 | Interventions and assessment tools addressing key concepts people need to know to appraise claims about treatment effects: a systematic mapping review | Mapping review |
| 46 | Evidence map of studies evaluating methods for conducting, interpreting and reporting overviews of systematic reviews of interventions: rationale and design | Mapping review |
| 47 | Mapping the systematic review toolbox | Mapping review |
| 48 | Heterogeneity of studies in anesthesiology systematic reviews: a meta-epidemiological review and proposal for evidence mapping | Mapping review |
| 49 | Alternative Title: A Systematic Mapping on CASE Tools Usability Assessment | Mapping review |
| 50 | Technological media and development A systematic mapping study and research agenda | Mapping review |
| 51 | Systematic mapping of existing tools to appraise methodological strengths and limitations of qualitative research: first stage in the development of the CAMELOT tool | Mapping review |
| 52 | The Impact of Controlled Vocabularies on Requirements Engineering Activities: A Systematic Mapping Study | Mapping review |
| 53 | Recommender Systems based on Scientific Publications: A Systematic Mapping | Mapping review |
| 54 | Secondary studies in the academic context: A systematic mapping and survey | Mapping review |
| 55 | Application of systematic evidence mapping to assess the impact of new research when updating health reference values: A case example using acrolein | Mapping review |
| 56 | A Systematic Mapping Study of Software Usability Studies | Mapping review |
| 57 | Mapping global research on climate and health using machine learning (a systematic evidence map) | Mapping review |
| 58 | Systematic mapping of global research on climate and health: a machine learning review | Mapping review |
| 59 | A Systematic Mapping Literature Review of Ethics in Healthcare Simulation and its Methodological Feasibility | Mapping review |
| 60 | Optimizing our evidence map for cognitive-communication interventions: How it can guide us to better outcomes for adults living with acquired brain injury | Mapping review |
| 61 | 2017 Research on big data – a systematic mapping study | Mapping review |
| 62 | Art Therapy in Advanced Cancer. A Mapping Review of the Evidence | Mapping review |
| 63 | 2011 A systematic mapping study of software product lines testing | Mapping review |
| 64 | A systematic mapping study on research in anemia assessment with non-invasive devices | Mapping review |
| 65 | A systematic mapping literature review of ethics in healthcare simulation and its methodological feasibility | Mapping review |
| 66 | Evidence Mapping of the Treatments for Breast Cancer-related Lymphedema | Mapping review |
| 67 | Awareness support in distributed software development: A systematic review and mapping of the literature | Mapping review |
| 68 | A systematic map of medical data preprocessing in knowledge discovery. Comput. Methods Programs Biomed | Mapping review |
| 69 | The extent and coverage of current knowledge of connected health: Systematic mapping study | Mapping review |
| 70 | An evidence map of systematic reviews to inform interventions in prediabetes | Mapping review |
| 71 | Text mining and semantics: a systematic mapping study | Mapping review |
| 72 | A systematic mapping study on modeling for industry 4.0 | Mapping review |
| 73 | The impact of financial circumstances on engagement with post-16 learning: A systematic map of research (EPPI-Centre Review) | Mapping review |
| 74 | Using Mapping Studies in Software Engineering | Updated |
| 75 | Mapping review could be seen as a subtype of scoping review and differentiating between the action of mapping evidence and presentation of evidence as maps may be helpful: response to Khalil et al | Updated |
| 76 | Systematic Mapping Studies in Software Engineering | Updated |
| 77 | Clarifying differences between review designs and methods | Updated |

**Supplement Table 3. Terminology, focus research fields, and terminology variations in 68 included documents**

| **N.** | **1st Author** | **Year** | **Title** | **Type of study** | **Research field** | **Note (field)** | **Terminology** | **Terminology variations** |
| --- | --- | --- | --- | --- | --- | --- | --- | --- |
| 1 | David L. Katz | 2003 | The evidence base for complementary and alternative medicine: methods of Evidence Mapping with application to CAM | gudiance | Health science | NR | Evidence mapping, Evidence Map | Yes |
| 2 | ﻿Salina Bates | 2007 | Systematic Maps to support the evidence base in social care | gudiance | Social Welfare | NR | Systematic map, Systematic mapping | Yes |
| 3 | ﻿Sarah E. Hetrick | 2008 | Evidence mapping: illustrating an emerging methodology to improve evidence-based practice in youth mental health | gudiance | Health science | NR | ﻿Evidence mapping, Evidence map | Yes |
| 4 | ﻿Anne F. Parkhill | 2008 | Searches for evidence mapping: effective, shorter, cheaper | gudiance | Health science | NR | ﻿Evidence mapping, Evidence map | Yes |
| 5 | ﻿Maria J. Grant | 2009 | A typology of reviews: an analysis of 14 review types and associated methodologies | gudiance | NR | NA | ﻿Mapping review, Systematic map | Yes |
| 6 | Russell R | 2009 | Issues and Challenges in Conducting Systematic Reviews to Support Development of Nutrient Reference Values: Workshop Summary: Nutrition Research Series, Vol. 2. | gudiance | Health science | NR | ﻿Evidence mapping, Evidence map | Yes |
| 7 | Janet Clapton (SCIE) | 2009 | SCIE Systematic mapping guidance: Social Care Institute for Excellence. London: Social Care Institute for Excellence. | gudiance | Social Welfare | NR | Systematic mapping, Systematic map | Yes |
| 8 | ﻿Barbara A. Kitchenham | 2010 | Using mapping studies as the basis for further research-A participant-observer case study | gudiance | others | Software engineering | Mapping ﻿study | NR |
| 9 | Cynthia Lum | 2010 | The Evidence-Based Policing Matrix | gudiance | Crime & Justice | NR | Evidence-Based Policing Matrix | NR |
| 10 | ﻿Peter Bragge | 2011 | The Global Evidence Mapping Initiative: scoping research in broad topic areas | gudiance | Health science | NR | ﻿Evidence mapping, Evidence map | Yes |
| 11 | ﻿李 伦 | 2011 | 一种新的证据总结方法--证据图简介 | gudiance | Health science | NR | ﻿Evidence mapping | NR |
| 12 | ﻿C. Schmucker | 2013 | Methods of evidence mapping. A systematic review | methodological study | Health science | NR | ﻿Evidence mapping, Evidence map | Yes |
| 13 | Birte Snilstveit | 2013 | Evidence Gap Maps — A Tool for Promoting Evidence-Informed Policy and Prioritizing Future Research | gudiance | NR | NA | Evidence gap map | Yes |
| 14 | Madeleine C. McKinnon. | 2015 | Sustainability: Map the evidence | gudiance | Social Welfare | NR | Evidence map | NR |
| 15 | Kai Petersen | 2015 | Guidelines for conducting systematic mapping studies in software engineering: An update | gudiance | others | Software engineering | ﻿Systematic mapping ﻿study | NR |
| 16 | Andrew Booth | 2015 | EVIDENT Guidance for Reviewing the Evidence: a compendium of methodological literature and websites | gudiance | NR | NA | Mapping Review, Mapping study | Yes |
| 17 | Helen R. Bayliss | 2016 | Updating and amending systematic reviews and systematic maps in environmental management | gudiance | Climate Solutions | Environmental management | Systematic map | NR |
| 18 | Barbara Buchberger | 2016 | Evidence mapping for decision making: feasibility versus accuracy - when to abandon high sensitivity in electronic searches | gudiance | Health science | NR | Evidence mapping | NR |
| 19 | Diane Cooper | 2016 | What is a "mapping study?" | gudiance | NR | NA | Mapping study, Systematic mapping review, Mapping research | Yes |
| 20 | ﻿Neal R. Haddaway | 2016 | The benefits of systematic mapping to evidence-based environmental management | methodological study | Climate Solutions | NR | Systematic map, ﻿Systematic mapping | Yes |
| 21 | Katy L. James | 2016 | A methodology for systematic mapping in environmental sciences | gudiance | Climate Solutions | Environmental sciences | Systematic mapping, Systematic map, Evidence gap map, Evidence mapping | Yes |
| 22 | Isomi M. Miake-Lye | 2016 | What is an evidence map? A systematic review of published evidence maps and their definitions, methods, and products | methodological study | NR | NA | Evidence mapping, Evidence map | Yes |
| 23 | Eva A Rehfuess | 2016 | An approach for setting evidence-based and stakeholder-informed research priorities in low- and middle-income countries | gudiance | NR | NA | Evidence map | NR |
| 24 | Birte Snilstveit | 2016 | Evidence & Gap Maps: A tool for promoting evidence informed policy and strategic research agendas | gudiance | NR | NA | Evidence and Gap Map | Yes |
| 25 | Carol L. Perryman | 2016 | Mapping studies | gudiance | NR | NA | Mapping Review, Mapping study | Yes |
| 26 | Department of Planning, Monitoring and Evaluation (DPME) | 2016 | Department of Planning, Monitoring and Evaluation (DPME), South Africa. 2016, Evidence Mapping_Policy Relevant Evidence Maps. | gudiance | others | Public sector | Evidence mapping, Evidence map, Systematic map, 3ie map | Yes |
| 27 | N. R. Haddaway | 2017 | A framework for stakeholder engagement during systematic reviews and maps in environmental management | gudiance | Climate Solutions | Environmental sciences | Systematic map | NR |
| 28 | Bethan C. O’Leary | 2017 | Evidence maps and evidence gaps: evidence review mapping as a method for collating and appraising evidence reviews to inform research and policy | gudiance | Climate Solutions | Environmental sciences | Evidence review mapping, Evidence review map | Yes |
| 29 | Birte Snilstveit (3ie) | 2017 | 3ie Evidence gap maps: a starting point for strategic evidence production and use, 3ie Working Paper 28. | gudiance | International Development | NR | 3ie Evidence gap maps, Evidence map, Systematic map | Yes |
| 30 | Neal R. Haddaway | 2018 | ROSES RepOrting standards for Systematic Evidence Syntheses: pro forma, flow-diagram and descriptive summary of the plan and conduct of environmental systematic reviews and systematic maps | gudiance | Climate Solutions | Environmental sciences | Systematic mapping, Systematic map | Yes |
| 31 | Christian Kohl | 2018 | Online tools supporting the conduct and reporting of systematic reviews and systematic maps: a case study on CADIMA and review of existing tools | gudiance | Climate Solutions | Environmental sciences | Systematic map | NR |
| 32 | Ashrita Saran | 2018 | Evidence and gap maps: a comparison of different approaches | methodological study | NR | NA | Evidence and gap map | Yes |
| 33 | Shannon Simonovich | 2018 | Re-Envisioning Evidence Gap Maps With Qualitative Research | gudiance | NR | NA | Evidence Gap Map | NR |
| 34 | Andrea C. Tricco | 2018 | PRISMA Extension for Scoping Reviews (PRISMA-ScR): Checklist and Explanation | gudiance | NR | NA | Evidence map | NR |
| 35 | Fares Alahdab | 2018 | Evidence maps: a tool to guide research agenda setting | gudiance | NR | NA | Evidence maps | NR |
| 36 | Howard White | 2018 | Campbell EGM reporting standards checklist | gudiance | others | Social science | Evidence and gap map | NR |
| 37 | Caroline Bradbury-Jones | 2019 | Advancing the science of literature reviewing in social research: the focused mapping review and synthesis | gudiance | others | Social science | Focused Mapping Review and Synthesis (FMRS). | Yes |
| 38 | Juleen Lam | 2019 | Low-calorie sweeteners and health outcomes: A demonstration of rapid evidence mapping (rEM) | gudiance | Health science | NR | Rapid Evidence Mapping, Evidence map | Yes |
| 39 | Taylor A.M.Wolffe | 2019 | Systematic evidence maps as a novel tool to support evidence-based decision-making in chemicals policy and risk management | gudiance | Climate Solutions | Environmental management | Systematic evidence map, Systematic evidence mapping | Yes |
| 40 | 李沐阳 | 2019 | 证据图检索实施情况与证据总结方法调查分析 | methodological study | NR | NA | Evidence mapping | NR |
| 41 | 田金徽 | 2019 | 证据图撰写注意事项 | gudiance | NR | NA | Evidence mapping | NR |
| 42 | Anthea Sutton | 2019 | Meeting the review family: exploring review types and associated information retrieval requirements | gudiance | NR | NA | Mapping Review, Evidence Map, Systematic Map, Systematic Mapping Review | Yes |
| 43 | David Gough | 2019 | Clarifying differences between reviews within evidence ecosystems | gudiance | NR | NA | Systematic Map, Map of maps | Yes |
| 44 | Adriana Mihaela Soaita | 2019 | A methodological quest for systematic literature mapping | gudiance | Social Welfare | NR | Systematic literature mapping, Systematic map | Yes |
| 45 | Carmen Cecilia Delgado Reyes | 2019 | Guía metodológica para la construcción de Mapas de Brechas de Evidencia. | gudiance | others | Social policy | Mapas de Brechas de Evidencia (Evidence gap map) | NR |
| 46 | Benjamin E. Nye | 2020 | Trialstreamer: Mapping and Browsing Medical Evidence in Real-Time | gudiance | Health science | NR | ﻿Evidence mapping, Evidence map | Yes |
| 47 | Howard White | 2020 | Guidance for producing a Campbell evidence and gap map | gudiance | others | Social science | Evidence and gap map; Mega-map; Map of maps | Yes |
| 48 | Daniele Wikoff | 2020 | Facilitation of risk assessment with evidence-based methods - A framework for use of systematic mapping and systematic reviews in determining hazard, developing toxicity values, and characterizing uncertainty | gudiance | Climate Solutions | Environmental management | Systematic map, Systematic evidence mapping | Yes |
| 49 | Taylor A M Wolffe | 2020 | A Survey of Systematic Evidence Mapping Practice and the Case for Knowledge Graphs in Environmental Health and Toxicology | methodological study | Climate Solutions | Environmental management | Systematic evidence map, Systematic evidence mapping | Yes |
| 50 | 李艳飞 | 2020 | 证据图谱的制作与报告 | gudiance | NR | NA | Eevidence mapping, Evidence map, Gap map | Yes |
| 51 | 李艳飞 | 2020 | 证据图谱方法及其在公共卫生领域的应用研究 | gudiance | others | Public health | Evidence mapping, Evidence map, Evidence and gap map; Mega-map; Map of maps | Yes |
| 52 | Ashrita Saran | 2020 | Evidence and gap maps | gudiance | others | Social science | Evidence and gap maps | NR |
| 53 | Jon Brassey | 2021 | Developing a fully automated evidence synthesis tool for identifying, assessing and collating the evidence | gudiance | Health science | NR | Evidence mapping, Evidence map | Yes |
| 54 | Diego Chambergo-Michilot | 2021 | [Scoping reviews, umbrella reviews and focused mapping review synthesis: methodological aspects and applications] | gudiance | NR | NA | Focused mapping review synthesis | NR |
| 55 | Thomas B. Røst | 2021 | Using neural networks to support high-quality evidence mapping | gudiance | NR | NA | Evidence mapping, Evidence map | Yes |
| 56 | Bastián Schuller-Martínez | 2021 | Graphical representation of the body of the evidence: the essentials for understanding the evidence gap map approach | gudiance | NR | NA | Evidence gap map | NR |
| 57 | Ian Shemilt | 2021 | Using automation to produce a ‘living map’ of the COVID-19 research literature | gudiance | Health science | NR | Evidence map | NR |
| 58 | Christian A. Candela-Uribe | 2021 | SMS-Builder: An adaptive software tool for building systematic mapping studies | gudiance | others | Software engineering | Systematic mapping studies | NR |
| 59 | Howard White | 2021 | The strategic use of evidence and gap maps to build evidence architecture | gudiance | NR | NA | Evidence and gap maps, Evidence map, Evidence mapping | Yes |
| 60 | Hanan Khalil | 2022 | Differentiating between mapping reviews and scoping reviews in the evidence synthesis ecosystem | gudiance | NR | NA | Mapping reviews | NR |
| 61 | Gustavo Navas | 2022 | Glaserian Systematic Mapping Study: An Integrating Methodology | gudiance | others | Software engineering | Systematic Mapping Study, Glaserian systematic mapping study | NR |
| 62 | Kristina A. Thayer | 2022 | Systematic evidence map (SEM) template: Report format and methods used for the US EPA Integrated Risk Information System (IRIS) program, Provisional Peer Reviewed Toxicity Value (PPRTV) program, and other "fit for purpose" literature-based human health an | gudiance | Climate Solutions | Environmental health field | Systematic evidence map | NR |
| 63 | ERNO VANHALA | 2022 | The Application Domains of Systematic Mapping Studies: A Mapping Study of the First Decade of Practice With the Method | methodological study | others | Software engineering | Systematic mapping study | NR |
| 64 | Andrew S Pullin(CEE) | 2022 | Collaboration for Environmental Evidence. Guidelines and standards for evidence synthesis in environmental management Version 5.1. Pullin A, Frampton G, Livoreil B, Petrokofsky G, editors. 2022.? | gudiance | Climate Solutions | Environmental sciences | Systematic map | NR |
| 65 | Fiona Campbell1 | 2023 | Mapping reviews, scoping reviews, and evidence and gap maps (EGMs): the same but different- the "Big Picture" review family | gudiance | NR | NA | Mapping review, evidence and gap map | Yes |
| 66 | Joshua R. Polanin | 2023 | Evidence Gap Maps in Education Research | gudiance | Education | NR | Evidence gap map | NR |
| 67 | Emily South | 2023 | Data visualisation in scoping reviews and evidence maps on health topics: a cross-sectional analysis | methodological study | Health science | NR | Evidence map, Mapping review, Systematic map | Yes |
| 68 | Hanan Khalil | 2023 | Advancing the methodology of mapping reviews: A scoping review | methodological study | NR | NA | Mapping review | NR |

*NR: Not Reported; NA: Not Applicable

**Supplement Table 4. Components and objectives of 55 definitions**

| **N.** | **Definitions** | **Research field** | **Systematic** | **Type of evidence** | **Content** | **Structure** | **Transparent** | **Visual display** | **Descriptive report** | **Users** | **Current state of research** | **Where evidence exists** | **Where it is lacking** |
| --- | --- | --- | --- | --- | --- | --- | --- | --- | --- | --- | --- | --- | --- |
| 1 | 1.Evidence mapping is a means of systematically organizing the base of evidence pertaining to a broad topic within medicine or public health so that the distribution, breadth, depth, methodology, and overall quality of pertinent evidence is characterized and made readily accessible. 2.A less systematic but nonetheless replicable process termed ‘mapping’ is an emerging concept that allows an understanding of the ‘extent, distribution and methodological quality of research’ relevant to broad topics. | Health science | 1 | 0 | 1 | 1 | 0 | 1 | 0 | 0 | 1 | 0 | 0 |
| 2 | Evidence mapping is a methodology that provides a comprehensive summary of the extent and distribution of the evidence in a broad clinical area, allowing a snapshot of where evidence exists and where it is lacking. | Health science | 0 | 0 | 1 | 1 | 0 | 1 | 1 | 0 | 1 | 1 | 1 |
| 3 | Evidence mapping describes the quantity, design and characteristics of research in broad topic areas, in contrast to systematic reviews, which usually address narrowly-focused research questions. The breadth of evidence mapping helps to identify evidence gaps, and may guide future research efforts. | Health science | 0 | 0 | 1 | 1 | 0 | 0 | 1 | 0 | 1 | 0 | 1 |
| 4 | 1.Evidence mapping is a method to facilitate exploring new ideas and hypotheses. It can be used to direct limited resources to potentially more fruitful areas for systematic review as well as to complement comprehensive systematic reviews on specific key questions. It aims to provide investigators with information about the type and amount of research available, the characteristics of that research, and the topics where a sufficient amount of evidence has accumulated for synthesis. | Health science | 1 | 0 | 1 | 1 | 0 | 0 | 0 | 1 | 1 | 0 | 1 |
| 5 | 证据图(Evidence Mapping)最早源于美国耶鲁大学系统评价补充与替代医学相关证据的研究,是将全面查找、科学分析、系统总结、高度概括、准确展示补充与替代医学研究全貌的方法称为Evidence Mapping | Health science | 1 | 0 | 1 | 0 | 0 | 1 | 1 | 0 | 1 | 0 | 0 |
| 6 | Evidence mapping is a relatively new approach to systematic evidence mapping, which is currently mainly used internationally. An overview is generated, with the help of which you can find out about the current state of research on therapeutic interventions or diagnostic measures for defined diseases or public health measures. | Health science | 1 | 0 | 1 | 1 | 0 | 0 | 1 | 0 | 1 | 0 | 0 |
| 7 | The term evidence mapping describes a system targeting an overview of the extent, nature and characteristics of a research field | Health science | 1 | 0 | 1 | 0 | 0 | 0 | 1 | 0 | 1 | 0 | 0 |
| 8 | 证据图是一种通过系统查找、科学分析、全面总结以及准确展示研究全貌的方法，能够描述研究数量和特征并指出研究的不足之处。证据图可用于评价临床证据的新兴概念，它比系统评价/Meta 分析更具包容性，对后续研究的开展也有很大的指导意义 | NR | 1 | 0 | 1 | 1 | 0 | 1 | 1 | 0 | 1 | 1 | 1 |
| 9 | 证据图研究通过全面检 索所关注问题的相关研究，系统总结相关研究的基本特征及结果，采用恰当的方式呈现该领域已有的证据、进展及存在的问题，为证据使用者提供所关注问题领域研究的全貌 | NR | 1 | 0 | 1 | 1 | 0 | 1 | 1 | 1 | 1 | 1 | 1 |
| 10 | 证据图谱是系统收集相关研究领域的现有证据、进行综合分析、科学评价、整合凝练、简明直观地呈现其研究现状、存在问题、发展方向和证据差距的一种新型证据综合研究方法。 | NR | 1 | 0 | 1 | 1 | 0 | 1 | 1 | 0 | 1 | 1 | 1 |
| 11 | 证据图谱是系统收集相关研究领域的现有证据、进行综合分析、科学评价，整合凝练、简明直观 地呈现其研究现状、存在问题、发展方向和证据差距的一种新型证据综合研究方法。 | others | 1 | 0 | 1 | 1 | 0 | 1 | 1 | 0 | 1 | 1 | 1 |
| 12 | Evidence mapping is a novel method of evidence synthesis that has received increased attention in recent years. Evidence mapping aims to transparently assess and structure what evidence has been generated in relation to a specific research question in order to identify patterns and gaps in the evidence-base. | others | 0 | 0 | 1 | 1 | 1 | 0 | 0 | 0 | 1 | 1 | 1 |
| 13 | Mapping is an evidence synthesis approach that aims to describe what research evidence is available that is relevant to a particular research or policy question. | NR | 1 | 0 | 1 | 0 | 0 | 0 | 0 | 0 | 1 | 0 | 0 |
| 14 | An evidence map is an overview of a broad research field that describes the volume, nature, and characteristics of research in that field | Health science | 0 | 0 | 1 | 1 | 0 | 0 | 1 | 0 | 1 | 0 | 0 |
| 15 | Evidence maps can show at a glance which areas or relationships have been studied most — whether it be the impact of ecotourism on local economies or of education on reducing harmful fishing practices. They can also highlight key gaps in the evidence base, and so guide the prioritization of research. | Social Welfare | 0 | 0 | 1 | 0 | 0 | 0 | 0 | 0 | 1 | 0 | 1 |
| 16 | Evidence map is a systematic search of a broad field to identify gaps in knowledge and/or future research needs that presents results in a user-friendly format, often a visual figure or graph, or a searchable database. | NR | 1 | 0 | 1 | 0 | 0 | 1 | 0 | 1 | 1 | 0 | 1 |
| 17 | Evidence mpas share similarities with scoping reviews and involve a systematic search of a body of literature to identify knowledge gaps, with a visual representation of results (such as a figure or graph). | NR | 1 | 0 | 0 | 0 | 0 | 1 | 0 | 0 | 1 | 0 | 1 |
| 18 | One of the main definitions of evidence maps is that they are the systematic organisation and illustration of a broad field of research evidence with the intent to characterise the breadth, depth and methodology of relevant evidence and identify gaps. Other times, an evidence map was defined as an approach to providing a visual representation and critical assessment of evidence landscape for a particular topic or question. A more recent definition was drawn from the published evidence maps in the literature and found it to be a systematic search of a broad field to identify gaps in knowledge and future research needs. | NR | 1 | 0 | 1 | 0 | 0 | 1 | 1 | 0 | 1 | 1 | 1 |
| 19 | Evidence map can be used to identify evidence gaps and present them in a user-friendly (and often visual) way | Health science | 0 | 0 | 1 | 0 | 0 | 1 | 0 | 0 | 1 | 0 | 1 |
| 20 | An evidence and gap map is a systematic [visual] presentation of the availability of relevant evidence [of effects] for a particular policy domain. The evidence is identified by a search following a pre-specified, published search protocol. [The map may be accompanied by a descriptive report to summarize the evidence for stakeholders such as researchers, research commissioners, policy makers, and practitioners] [Evidence maps summarize what evidence there is, not what the evidence says] | NR | 1 | 0 | 1 | 1 | 1 | 1 | 1 | 1 | 1 | 0 | 0 |
| 21 | Evidence and Gap Maps (EGMs) are a systematic evidence synthesis product which display the available evidence relevant to a specific research question. | others (Social science) | 1 | 0 | 1 | 0 | 0 | 0 | 0 | 0 | 1 | 0 | 0 |
| 22 | Evidence gap maps can be defined as thematic collections of evidence structured around a framework that graphically and schematically represents the types of interventions and outcomes relevant to a particular problem | NR | 0 | 1 | 0 | 1 | 0 | 1 | 0 | 0 | 1 | 0 | 0 |
| 23 | Evidence and gap maps are described as “a systematic presentation of all relevant evidence of a specified kind for a particular sector, sub-sector, or geography”. Evidence and gap maps are a systematic evidence synthesis product which displays the available evidence relevant to a specific research question. Evidence and gap maps consist of primary dimensions or framework (rows and columns) and secondary dimensions or filters, enabling exploration of the map using a particular focus (e.g., looking at particular populations or study designs). It creates a visual, web-based, and interactive output | NR | 1 | 0 | 1 | 1 | 0 | 1 | 0 | 0 | 1 | 0 | 0 |
| 24 | An Evidence Gap Map (EGM), incorporated as a part of systematic review, is an ideal translation and presentation technique because it can provide audiences with an understanding of the state of the field’s literature in an intuitive fashion. | Education | 0 | 0 | 1 | 0 | 0 | 1 | 0 | 1 | 1 | 0 | 0 |
| 25 | Gap Evidence Maps (GEMs) are a new tool designed to identify criteria that facilitate decision-making in monitoring, evaluation, and policy formulation. GEMs provide an indication of (i) the most relevant interventions carried out and considered for a sector, (ii) the key outcome variables of these interventions, and (iii) the evaluations and results obtained from the relationship between interventions and outcome variables. | others (Social policy) | 0 | 1 | 1 | 1 | 0 | 0 | 0 | 0 | 1 | 0 | 0 |
| 26 | 3ie EGMs are collections of evidence on the effects of development policies and programmes in a particular sector or thematic area | International Development | 0 | 0 | 1 | 0 | 0 | 0 | 0 | 1 | 1 | 0 | 0 |
| 27 | Evidence gap maps are evidence collections that map out existing and ongoing systematic reviews or primary studies in a sector or subsector, such as maternal health, HIV/AIDS and agriculture. Theypresent a visual overview of existing evidence using a framework of policy relevant interventions and outcomes, and provide access to userfriendly summaries of the -included studies | NR | 0 | 1 | 1 | 1 | 0 | 1 | 0 | 0 | 1 | 1 | 0 |
| 28 | We present a new approach, evidence review mapping, designed to produce a visual representation and critical assessment of the review landscape for a particular environmental topic or question. | Climate Solutions | 0 | 1 | 1 | 0 | 0 | 1 | 0 | 0 | 1 | 0 | 0 |
| 29 | The Evidence-Based Policing Matrix is a research-to-practice translation tool that organizes moderate to very rigorous evaluations of police interventions visually, allowing agencies and researchers to view the field of research in this area. | Crime & Justice | 0 | 1 | 1 | 1 | 0 | 1 | 0 | 1 | 1 | 1 | 0 |
| 30 | Focused Mapping Review and Synthesis (FMRS) is a new form of review. The aim of the FMRS is to address epistemological questions that relate to a particular research field. There are four key features of FMRS. It: (1) Focuses on a defined field of knowledge rather than a body of evidence; (2) Creates a descriptive map or topography of key features of research within the field rather than a synthesis of findings; (3) Comments on the overall approach to knowledge production rather than the state of the evidence; and (4) Examines this within a broader epistemological context. | others | 0 | 0 | 1 | 1 | 0 | 1 | 0 | 0 | 1 | 0 | 0 |
| 31 | This new proposal was presented by Bradbury-Jones et al, who define it based on four main characteristics: 1) focusing on a defined field of knowledge instead of the body of evidence; 2) mapping the key features of research in that field rather than synthesizing the content; 3) commenting on the overall approach to scientific production rather than the state of evidence; and 4) examining from an epistemological perspective. FMRS is useful for questions where classical mapping (synthesis focused on map review) would be unfeasible due to addressing too many articles (9). They are also useful because the flexibility of their design allows them to answer a variety of specific questions that arise from complex areas of knowledge | NR | 0 | 0 | 1 | 1 | 0 | 1 | 0 | 0 | 1 | 0 | 0 |
| 32 | 1.A resource-efficient form of knowledge synthesis where components of the review process are simplified to produce a visual and quantitative representation of the scientific evidence from which to commission further reviews and/or primary research by identifying gaps in research. | Health science | 0 | 0 | 1 | 0 | 0 | 1 | 0 | 0 | 1 | 0 | 1 |
| 33 | Systematic mapping studies or scoping studies are designed to give an overview of a research area through classification and counting contributions in relation to the categories of that classification. It involves searching the literature in order to know what topics have been covered in the literature, and where the literature has been published | others | 0 | 0 | 1 | 0 | 0 | 0 | 1 | 0 | 1 | 1 | 0 |
| 34 | A Systematic Mapping Study is an instrument frequently used to carry out a search process, identification, and classification of studies in different fields. | others | 0 | 0 | 1 | 1 | 0 | 0 | 0 | 0 | 1 | 0 | 0 |
| 35 | The Systematic Mapping Study (SMS) is a rigorous review process of the scientific literature. | others | 0 | 0 | 1 | 0 | 0 | 0 | 0 | 0 | 0 | 0 | 0 |
| 36 | Glaserian Systematic Mapping Study (GSMS) is a methodology that combines SMS and Glaserian Grounded Theory (GGT) | others | 0 | 0 | 1 | 0 | 0 | 0 | 0 | 0 | 0 | 0 | 0 |
| 37 | Mapping studies use the same basic methodology as SLRs but aim to identify and classify all research related to a broad software engineering topic rather than answering questions about the relative merits of competing technologies that conventional SLRs address. They are intended to provide an overview of a topic area and identify whether there are sub-topics with sufficient primary studies to conduct conventional SLRs and also to identify sub-topics where more primary studies are needed. | others | 0 | 0 | 1 | 0 | 0 | 0 | 0 | 0 | 1 | 0 | 1 |
| 38 | Systematic evidence maps (SEMs) provide a broad and comprehensive overview of an evidence base. They facilitate the identification of trends which can be used to inform more efficient systematic review, or more targeted primary research. | Climate Solutions | 0 | 0 | 1 | 0 | 0 | 0 | 1 | 0 | 1 | 0 | 1 |
| 39 | A queryable database of systematically gathered evidence (eg, academic literature and industry reports). SEMs extract and structure data and/or metadata for exploration following a rigorous methodology which aims to minimize bias and maximize transparency. | Climate Solutions | 1 | 0 | 1 | 0 | 0 | 0 | 0 | 0 | 1 | 0 | 0 |
| 40 | Systematic Evidence Maps (SEMs) are proving to be a particularly valuable analysis tool to inform the scope of complex human health assessments | Climate Solutions | 0 | 0 | 1 | 0 | 0 | 0 | 0 | 0 | 1 | 0 | 0 |
| 41 | A time-limited, systematic search for literature related to a well-defined but still broad academic theme whose parameters and limits are openly set from the start; and the exploration and synthesis of key temporal, geographical, conceptual and thematic features of this literature. | Social Welfare | 1 | 0 | 1 | 1 | 0 | 0 | 0 | 0 | 1 | 0 | 0 |
| 42 | 2.Systematic maps gather together existing literature in a specific topic area and categorise it according to predefined keywords to create a coded database of literature. The topic area can be broad or narrow depending on the needs of the project in question | Social Welfare | 0 | 0 | 1 | 1 | 0 | 1 | 0 | 0 | 1 | 0 | 0 |
| 43 | Systematic maps may characterize studies in other ways such as according to theoretical perspective, population group or the setting within which studies were undertaken. | NR | 0 | 0 | 1 | 0 | 0 | 0 | 0 | 0 | 1 | 0 | 0 |
| 44 | The objectives of SMs and SRs are fundamentally similar; to collate and describe all of the available published research evidence on a topic in an objective, repeatable and transparent manner | Climate Solutions | 0 | 0 | 1 | 0 | 1 | 0 | 0 | 0 | 1 | 0 | 0 |
| 45 | Systematic maps rely on evidence-based methods to characterize the state of knowledge for a topic. The overall objective of the systematic maps involve a broad characterization of a topic, allowing for well-informed scoping and prioritization of outcomes and endpoints to be considered for SR. | Climate Solutions | 0 | 0 | 1 | 0 | 0 | 0 | 0 | 0 | 1 | 0 | 0 |
| 46 | A Systematic Map is an evidence synthesis method that aims to provide an accurate description of the evidence base relating to a particular question. | Climate Solutions | 0 | 0 | 1 | 0 | 0 | 0 | 0 | 0 | 1 | 0 | 0 |
| 47 | 1.A key issue is to explore the distribution of available knowledge before deciding how best it can be further used, for example, in systematic reviews. This process is known as systematic mapping. The methodology was originally developed by the EPPI-Centre and has been adapted by SCIE for use with social care topics in consultation with the EPPI-Centre. | Social Welfare | 0 | 0 | 1 | 0 | 0 | 0 | 0 | 0 | 1 | 0 | 0 |
| 48 | Systematic mapping was developed in social sciences in response to a lack of empirical data when answering questions using systematic review methods, and a need for a method to describe the literature across a broad subject of interest. Systematic mapping is a form of evidence synthesis. It is the method used to collect, collate, and present research evidence. | Climate Solutions | 0 | 0 | 1 | 1 | 0 | 1 | 0 | 0 | 1 | 0 | 0 |
| 49 | Systematic mapping has emerged as a very popular method for evidence synthesis, as a first step in the evidence synthesis pathway and as a means of highlighting knowledge clusters and gaps | Climate Solutions | 0 | 0 | 1 | 0 | 0 | 0 | 0 | 0 | 1 | 0 | 1 |
| 50 | One method of exploring the literature in a broad topic area is known as systematic mapping. | Social Welfare | 0 | 0 | 1 | 0 | 0 | 0 | 0 | 0 | 1 | 0 | 0 |
| 51 | Map out and categorize existing literature from which to commission further reviews and/or primary research by identifying gaps in research literature. Mapping reviews enable the contextualization of in-depth systematic literature reviews within broader literature and identification of gaps in the evidence base. They are a valuable tool in offering policymakers, practitioners and researchers an explicit and transparent means of identifying narrower policy and practice-relevant review questions. | NR | 0 | 0 | 1 | 1 | 1 | 0 | 0 | 1 | 1 | 0 | 1 |
| 52 | A mapping review is "a secondary study that reviews articles related to a specific research topic" ! lt has three principal objectives. i) to provide an overview of a research area to assess the existingevidence’, (il) to identify gaps in sets of primary studies, where new or better primary studies arerequired (ili) to pinpoint specific knowledge gaps where more complete systematic literature reviewsmight be required. | NR | 0 | 0 | 1 | 0 | 0 | 0 | 0 | 0 | 1 | 1 | 1 |
| 53 | Overall, mapping is a systematic approach to understanding the “map” of a profession, theory, research question, or practice. The term mapping is also used for “concept mapping,” which shows how concepts are related in a visual way. Similarly, mapping studies can show how literature is disseminated through journals, books, websites, and other channels. | NR | 1 | 0 | 1 | 0 | 0 | 0 | 0 | 0 | 1 | 0 | 0 |
| 54 | Mapping reviews are also a transparent, rigorous, and systematic approach to identifying, describing, and cataloging evidence and evidence gaps in a broader topic area. Tey are to collate, describe, and catalog the available evidence relating to the question of interest. | NR | 1 | 0 | 1 | 1 | 1 | 0 | 0 | 0 | 1 | 1 | 1 |
| 55 | Mapping studies are also reviews, but they do not discuss the findings. They are based on the concept that published articles not only represent findings,but, indirectly , represent activity related to the finding.It is a review that seeks to identify, not results, but linkages. Mapping focuses on characteristics such as where the activity took place, where the funding came from, and in what journal or other medium it was presented. Mapping often focuses on published items but need not; some mapping studies include other media. | NR | 0 | 0 | 1 | 0 | 0 | 0 | 0 | 0 | 1 | 0 | 0 |

*NR: Not Reported; 1: Yes; 0: No

**Supplement Table 5.** **Timeline for developing mapping reviews and comparison with other methods**

| **N.** | **1st Author** | **Year** | **Timeline** | **Mapping VS Systematic review** | **Mapping VS Scoping review** |
| --- | --- | --- | --- | --- | --- |
| 1 | David L. Katz | 2003 | NR | Yes | No |
| 2 | ﻿Salina Bates | 2007 | 6-12months | Yes | No |
| 3 | ﻿Sarah E. Hetrick | 2008 | 2 years | Yes | No |
| 4 | ﻿Anne F. Parkhill | 2008 | NR | No | No |
| 5 | ﻿Maria J. Grant | 2009 | NR | No | Yes |
| 6 | Russell R | 2009 | NR | No | No |
| 7 | Janet Clapton (SCIE) | 2009 | over 12months | No | No |
| 8 | ﻿Barbara A. Kitchenham | 2010 | NR | Yes | Yes |
| 9 | Cynthia Lum | 2010 | NR | No | No |
| 10 | ﻿Peter Bragge | 2011 | There are many resource-intensive steps in evidence mapping. Preliminary calculations performed by the GEM Initiative, based on time spent to develop detailed search strategies, search databases and select studies, indicate a resource cost of 1.5 minutes for each citation reviewed | Yes | Yes |
| 11 | ﻿李 伦 | 2011 | NR | Yes | No |
| 12 | ﻿C. Schmucker | 2013 | 1-6months | Yes | Yes |
| 13 | Birte Snilstveit | 2013 | 2-3m; 2 Years; 6m-1year | Yes | Yes |
| 14 | Madeleine C. McKinnon. | 2015 | NR | No | No |
| 15 | Kai Petersen | 2015 | NR | Yes | Yes |
| 16 | Andrew Booth | 2015 | 1-4 months | Yes | Yes |
| 17 | Helen R. Bayliss | 2016 | NR | No | No |
| 18 | Barbara Buchberger | 2016 | NR | No | No |
| 19 | Diane Cooper | 2016 | NR | No | Yes |
| 20 | ﻿Neal R. Haddaway | 2016 | NR | Yes | No |
| 21 | Katy L. James | 2016 | NR | Yes | Yes |
| 22 | Isomi M. Miake-Lye | 2016 | NR | No | Yes |
| 23 | Eva A Rehfuess | 2016 | NR | No | No |
| 24 | Birte Snilstveit | 2016 | less than 6 months | Yes | Yes |
| 25 | Carol L. Perryman | 2016 | NR | Yes | Yes |
| 26 | Department of Planning, Monitoring and Evaluation (DPME) | 2016 | 6 months | Yes | Yes |
| 27 | N. R. Haddaway | 2017 | NR | No | No |
| 28 | Bethan C. O’Leary | 2017 | NR | No | No |
| 29 | Birte Snilstveit (3ie) | 2017 | 3-6months | Yes | No |
| 30 | Neal R. Haddaway | 2018 | NR | No | No |
| 31 | Christian Kohl | 2018 | NR | No | No |
| 32 | Ashrita Saran | 2018 | 3-6 months | Yes | No |
| 33 | Shannon Simonovich | 2018 | NR | No | No |
| 34 | Andrea C. Tricco | 2018 | NR | No | Yes |
| 35 | Fares Alahdab | 2018 | NR | No | No |
| 36 | Howard White | 2018 | NR | No | No |
| 37 | Caroline Bradbury-Jones | 2019 | NR | No | No |
| 38 | Juleen Lam | 2019 | NR | No | No |
| 39 | Taylor A.M.Wolffe | 2019 | NR | Yes | Yes |
| 40 | 李沐阳 | 2019 | NR | Yes | No |
| 41 | 田金徽 | 2019 | NR | No | No |
| 42 | Anthea Sutton | 2019 | NR | No | Yes |
| 43 | David Gough | 2019 | NR | Yes | Yes |
| 44 | Adriana Mihaela Soaita | 2019 | NR | No | No |
| 45 | Carmen Cecilia Delgado Reyes | 2019 | 6 months | No | No |
| 46 | Benjamin E. Nye | 2020 | NR | No | No |
| 47 | Howard White | 2020 | 6-12months | No | No |
| 48 | Daniele Wikoff | 2020 | NR | Yes | No |
| 49 | Taylor A M Wolffe | 2020 | NR | No | No |
| 50 | 李艳飞 | 2020 | NR | Yes | No |
| 51 | 李艳飞 | 2020 | NR | Yes | No |
| 52 | Ashrita Saran | 2020 | NR | No | No |
| 53 | Jon Brassey | 2021 | NR | No | No |
| 54 | Diego Chambergo-Michilot | 2021 | NR | No | Yes |
| 55 | Thomas B. Røst | 2021 | NR | No | No |
| 56 | Bastián Schuller-Martínez | 2021 | NR | Yes | Yes |
| 57 | Ian Shemilt | 2021 | NR | No | No |
| 58 | Christian A. Candela-Uribe | 2021 | NR | No | No |
| 59 | Howard White | 2021 |  | No | No |
| 60 | Hanan Khalil | 2022 | NR | Yes | Yes |
| 61 | Gustavo Navas | 2022 | NR | No | No |
| 62 | Kristina A. Thayer | 2022 | NR | No | No |
| 63 | ERNO VANHALA | 2022 | NR | Yes | Yes |
| 64 | Andrew S Pullin(CEE) | 2022 | NR | Yes | No |
| 65 | Fiona Campbell1 | 2023 | NR | No | Yes |
| 66 | Joshua R. Polanin | 2023 | NR | Yes | No |
| 67 | Emily South | 2023 | NR | No | No |
| 68 | Hanan Khalil | 2023 | NR | No | No |

*NR: Not Reported

**Supplement Table 6. Potential reporting characteristics for title, author, abstract, and background of mapping reviews**

| **N.** | **Type of study** | **1st Author** | **Year** | **Title** | **Author** | **Abstract** | **Background** |
| --- | --- | --- | --- | --- | --- | --- | --- |
| 1 | gudiance | David L. Katz | 2003 | NR | NR | NR | NR |
| 2 | gudiance | ﻿Salina Bates | 2007 | NR | NR | NR | NR |
| 3 | gudiance | ﻿Sarah E. Hetrick | 2008 | NR | NR | NR | NR |
| 4 | gudiance | ﻿Anne F. Parkhill | 2008 | NR | NR | NR | NR |
| 5 | gudiance | ﻿Maria J. Grant | 2009 | NR | NR | NR | NR |
| 6 | gudiance | Russell R | 2009 | NR | NR | NR | NR |
| 7 | gudiance | Janet Clapton (SCIE) | 2009 | NR | NR | NR | Objectives: Aims of map: This section has core aims being the same across all maps; Specific map aims also to be highlighted. |
| 8 | gudiance | ﻿Barbara A. Kitchenham | 2010 | NR | NR | NR | NR |
| 9 | gudiance | Cynthia Lum | 2010 | NR | NR | NR | NR |
| 10 | gudiance | ﻿Peter Bragge | 2011 | NR | NR | NR | NR |
| 11 | gudiance | ﻿李 伦 | 2011 | NR | NR | NR | NR |
| 12 | methodological study | ﻿C. Schmucker | 2013 | NR | NR | NR | NR |
| 13 | gudiance | Birte Snilstveit | 2013 | NR | NR | NR | NR |
| 14 | gudiance | Madeleine C. McKinnon. | 2015 | NR | NR | NR | NR |
| 15 | gudiance | Kai Petersen | 2015 | NR | NR | NR | Rationale: Provide information on the background of the topic studied. Describe the need for the mapping, and highlight the usefulness. Provide an overview of existing secondary and tertiary studies in the area. |
| 16 | gudiance | Andrew Booth | 2015 | NR | NR | NR | NR |
| 17 | gudiance | Helen R. Bayliss | 2016 | NR | NR | NR | NR |
| 18 | gudiance | Barbara Buchberger | 2016 | NR | NR | NR | NR |
| 19 | gudiance | Diane Cooper | 2016 | NR | NR | NR | NR |
| 20 | methodological study | ﻿Neal R. Haddaway | 2016 | NR | NR | NR | NR |
| 21 | gudiance | Katy L. James | 2016 | NR | NR | NR | Rationale: Background and rationale for the systematic map as in systematic review. |
| 22 | methodological study | Isomi M. Miake-Lye | 2016 | NR | NR | NR | NR |
| 23 | gudiance | Eva A Rehfuess | 2016 | NR | NR | NR | NR |
| 24 | gudiance | Birte Snilstveit | 2016 | NR | NR | NR | NR |
| 25 | gudiance | Carol L. Perryman | 2016 | NR | NR | NR | NR |
| 26 | gudiance | Department of Planning, Monitoring and Evaluation (DPME) | 2016 | NR | NR | NR | NR |
| 27 | gudiance | N. R. Haddaway | 2017 | NR | NR | NR | NR |
| 28 | gudiance | Bethan C. O’Leary | 2017 | NR | NR | NR | NR |
| 29 | gudiance | Birte Snilstveit (3ie) | 2017 | NR | NR | NR | NR |
| 30 | gudiance | Neal R. Haddaway | 2018 | The title must indicate that it is a systematic map, and should indicate if it is an update/amendment: e.g. "…A systematic map update." The title should normally be the same or very similar to the review question. | The full names, institutional addresses and email addresses for all authors must be provided. | The abstract of the manuscript must not exceed 500 words and must be structured into separate sections: Background, the context and purpose of the review, including the review question; Methods, how the review was performed (specifically mention search strategy, inclusion criteria, critical appraisal (optional), meta-data extraction and coding, and narrative synthesis); Results, the main findings, including results of search and assessment of evidence base; Conclusions, brief summary and potential implications for policy/management and research. | Rationale & Objectives: Describe the rationale for the review in the context of what is already known. Reviews must indicate why this study was necessary and what it aims to contribute to the field. |
| 31 | gudiance | Christian Kohl | 2018 | NR | NR | NR | NR |
| 32 | methodological study | Ashrita Saran | 2018 | Define the scope of the map, which should be stated as a clear title | NR | NR | Objectives: Clearly determine the intended purpose of the planned evidence map, including the type of evidence to be included (e.g. effectiveness) and the planned structure of the map |
| 33 | gudiance | Shannon Simonovich | 2018 | NR | NR | NR | NR |
| 34 | gudiance | Andrea C. Tricco | 2018 | Identify the report as a scoping review (evidence map) | NR | Provide a structured summarythatincludes(as applicable):background, objectives, eligibility criteria, sources of evidence, charting methods, results, and conclusions that relate to the review questions and objectives. | Rationale: Describe the rationale for the review in the context of what is already known. Explain why the review questions/objectives lend themselves to a scoping review approach. Objectives: Provide an explicit statement of the questions and objectives being addressed with reference to their key elements (e.g., population or participants, concepts, and context) or other relevant key elements used to conceptualize the review questions and/or objectives. |
| 35 | gudiance | Fares Alahdab | 2018 | NR | NR | NR | NR |
| 36 | gudiance | Howard White | 2018 | Follow the standard Campbell EGM title template: The title of the EGM should define the scope of the map.  The long title provides further information such as the type of studies being included. The short title may be centred on population, outcomes or intervention. | List names and affiliations of all authors | Prepare a structured abstract to provide a succinct overview of the EGM. In the interests of brevity it is highly desirable for authors to provide an abstract of less than 700 words, and it should be no more than 1000 words in length. | Rationale: Provide a concise description of the scope of the EGM, and why it is important to do the EGM.  Objectives: State the objectives, where appropriate in a single concise sentence. State the types of evidence being shown in the EGM explicitly in the objectives |
| 37 | gudiance | Caroline Bradbury-Jones | 2019 | NR | NR | NR | NR |
| 38 | gudiance | Juleen Lam | 2019 | NR | NR | NR | NR |
| 39 | gudiance | Taylor A.M.Wolffe | 2019 | NR | NR | NR | NR |
| 40 | methodological study | 李沐阳 | 2019 | NR | NR | NR | NR |
| 41 | gudiance | 田金徽 | 2019 | NR | NR | NR | NR |
| 42 | gudiance | Anthea Sutton | 2019 | NR | NR | NR | NR |
| 43 | gudiance | David Gough | 2019 | NR | NR | NR | NR |
| 44 | gudiance | Adriana Mihaela Soaita | 2019 | NR | NR | NR | NR |
| 45 | gudiance | Carmen Cecilia Delgado Reyes | 2019 | NR | NR | NR | NR |
| 46 | gudiance | Benjamin E. Nye | 2020 | NR | NR | NR | NR |
| 47 | gudiance | Howard White | 2020 | The title of the EGM should define the scope of the map.  The long title provides further information such as the type of studies being included. The short title may be centred on population, outcomes or intervention. | NR | NR | Objectives: Maps can be used for various purposes, and the main purpose may vary by type of map |
| 48 | gudiance | Daniele Wikoff | 2020 | NR | NR | NR | NR |
| 49 | methodological study | Taylor A M Wolffe | 2020 | NR | NR | NR | NR |
| 50 | gudiance | 李艳飞 | 2020 | NR | NR | NR | 研究背景 (合理性和目的) |
| 51 | gudiance | 李艳飞 | 2020 | NR | NR | NR | NR |
| 52 | gudiance | Ashrita Saran | 2020 | The title of the EGMs should define the scope of the map. | NR | NR | Objectives: The type of evidence included in the EGM depends on the research questions. A mega-map includes only systematic reviews and EGMs, while a typical effectiveness map will include systematic review and impact evaluations. There is even a “map of maps” that includes only other EGMs |
| 53 | gudiance | Jon Brassey | 2021 | NR | NR | NR | NR |
| 54 | gudiance | Diego Chambergo-Michilot | 2021 | NR | NR | NR | NR |
| 55 | gudiance | Thomas B. Røst | 2021 | NR | NR | NR | NR |
| 56 | gudiance | Bastián Schuller-Martínez | 2021 | NR | NR | NR | NR |
| 57 | gudiance | Ian Shemilt | 2021 | NR | NR | NR | NR |
| 58 | gudiance | Christian A. Candela-Uribe | 2021 | NR | NR | NR | NR |
| 59 | gudiance | Howard White | 2021 | NR | NR | NR | Objectives: An approach to using evidence and gap maps to build evidence architecture |
| 60 | gudiance | Hanan Khalil | 2022 | NR | NR | NR | NR |
| 61 | gudiance | Gustavo Navas | 2022 | NR | NR | NR | NR |
| 62 | gudiance | Kristina A. Thayer | 2022 | NR | NR | NR | Rationale: The introduction should include a rationale on why the SEM is being developed and background on the topic.  Objectives: Specific aims as outlined by the Populations, Exposures, Comparators and Outcomes (PECO) criteria |
| 63 | methodological study | ERNO VANHALA | 2022 | NR | NR | NR | NR |
| 64 | gudiance | Andrew S Pullin(CEE) | 2022 | The title should express the review findings and indicate that it is a systematic map | The full names, institutional addresses, and email addresses for all authors must be included on the title page. The corresponding author should also be indicated. | The Abstract of the manuscript should not exceed 500 words and must be structured into the following separate sections: Background, the context and purpose of the review, including the review question; Methods, how the review was performed including brief overview of all methodological steps; Review findings, the main findings, including results of search and assessment of evidence base; Conclusions, brief summary and potential implications for policy/management and research. | Rationale & Objectives: The Background section should be written in a way that is accessible to readers without specialist knowledge in that area and must clearly state - and, if helpful, illustrate - the background to the review and its aims. Reports should indicate why this study was necessary and what it aimed to contribute to the field. A theory of change and/or conceptual model should be presented that links the intervention or exposure to the outcome. The role of commissioners and other stakeholders in the formulation of the question should be described and explained. The section should end with a brief statement of what is being reported in the article. A clear reference should be made to the protocol and any differences between what was planned and what was conducted. |
| 65 | gudiance | Fiona Campbell1 | 2023 | NR | NR | NR | NR |
| 66 | gudiance | Joshua R. Polanin | 2023 | NR | NR | NR | NR |
| 67 | methodological study | Emily South | 2023 | NR | NR | NR | NR |
| 68 | methodological study | Hanan Khalil | 2023 | NR | NR | NR | Objectives: Assessed the aims stated in the mapping review studies |

*NR: Not Reported

**Supplement Table 7. Potential reporting characteristics for methods of mapping reviews**

| **N.** | **Type of study** | **1st Author** | **Year** | **Stakeholders** | **Registration and protocol** | **Eligibility criteria** | **Search sources** | **Search strategy** | **Selection process** | **Data extraction and coding** | **Data collection process** | **Critical appraisal** | **Data presentation and analysis** |
| --- | --- | --- | --- | --- | --- | --- | --- | --- | --- | --- | --- | --- | --- |
| 1 | gudiance | David L. Katz | 2003 | Yes | NR | Yes | Yes | Yes | Yes | Yes | Yes | NR | Yes |
| 2 | gudiance | ﻿Salina Bates | 2007 | Yes | NR | Yes | Yes | Yes | Yes | Yes | Yes | NR | Yes |
| 3 | gudiance | ﻿Sarah E. Hetrick | 2008 | Yes | NR | Yes | Yes | Yes | Yes | Yes | Yes | Yes | Yes |
| 4 | gudiance | ﻿Anne F. Parkhill | 2008 | NR | NR | NR | Yes | Yes | NR | NR | NR | NR | NR |
| 5 | gudiance | ﻿Maria J. Grant | 2009 | NR | NR | NR | NR | NR | NR | NR | NR | NR | NR |
| 6 | gudiance | Russell R | 2009 | NR | NR | NR | NR | NR | NR | NR | NR | NR | NR |
| 7 | gudiance | Janet Clapton (SCIE) | 2009 | Yes | Yes | Yes | Yes | Yes | Yes | Yes | Yes | Yes | Yes |
| 8 | gudiance | ﻿Barbara A. Kitchenham | 2010 | NR | NR | NR | NR | NR | NR | NR | NR | NR | NR |
| 9 | gudiance | Cynthia Lum | 2010 | NR | NR | NR | NR | NR | NR | NR | NR | NR | NR |
| 10 | gudiance | ﻿Peter Bragge | 2011 | Yes | NR | Yes | Yes | Yes | Yes | Yes | Yes | NR | Yes |
| 11 | gudiance | ﻿李 伦 | 2011 | Yes | NR | Yes | Yes | Yes | NR | Yes | Yes | NR | Yes |
| 12 | methodological study | ﻿C. Schmucker | 2013 | Yes | NR | Yes | Yes | Yes | Yes | Yes | Yes | Yes | Yes |
| 13 | gudiance | Birte Snilstveit | 2013 | Yes | NR | Yes | Yes | Yes | Yes | Yes | NR | Yes | Yes |
| 14 | gudiance | Madeleine C. McKinnon. | 2015 | NR | NR | NR | NR | NR | NR | NR | NR | NR | NR |
| 15 | gudiance | Kai Petersen | 2015 | Yes | NR | Yes | Yes | Yes | Yes | Yes | Yes | Yes | Yes |
| 16 | gudiance | Andrew Booth | 2015 | NR | NR | NR | NR | NR | NR | NR | NR | NR | NR |
| 17 | gudiance | Helen R. Bayliss | 2016 | NR | NR | NR | NR | NR | NR | NR | NR | NR | Yes |
| 18 | gudiance | Barbara Buchberger | 2016 | NR | NR | NR | NR | Yes | NR | NR | NR | NR | NR |
| 19 | gudiance | Diane Cooper | 2016 | NR | NR | NR | NR | NR | NR | NR | NR | NR | NR |
| 20 | methodological study | ﻿Neal R. Haddaway | 2016 | NR | NR | NR | NR | NR | NR | NR | NR | NR | NR |
| 21 | gudiance | Katy L. James | 2016 | Yes | Yes | Yes | Yes | Yes | Yes | Yes | Yes | Yes | Yes |
| 22 | methodological study | Isomi M. Miake-Lye | 2016 | Yes | NR | NR | NR | NR | NR | NR | NR | NR | Yes |
| 23 | gudiance | Eva A Rehfuess | 2016 | NR | NR | Yes | Yes | Yes | Yes | Yes | Yes | NR | Yes |
| 24 | gudiance | Birte Snilstveit | 2016 | Yes | NR | Yes | Yes | Yes | Yes | Yes | NR | Yes | Yes |
| 25 | gudiance | Carol L. Perryman | 2016 | Yes | NR | Yes | Yes | NR | NR | NR | NR | NR | NR |
| 26 | gudiance | Department of Planning, Monitoring and Evaluation (DPME) | 2016 | Yes | NR | Yes | Yes | Yes | Yes | Yes | Yes | Yes | Yes |
| 27 | gudiance | N. R. Haddaway | 2017 | Yes | NR | NR | NR | NR | NR | NR | NR | NR | NR |
| 28 | gudiance | Bethan C. O’Leary | 2017 | Yes | NR | Yes | Yes | Yes | Yes | Yes | NR | Yes | Yes |
| 29 | gudiance | Birte Snilstveit (3ie) | 2017 | NR | NR | NR | NR | NR | NR | NR | NR | NR | NR |
| 30 | gudiance | Neal R. Haddaway | 2018 | Yes | Yes | Yes | Yes | Yes | Yes | Yes | Yes | Yes | Yes |
| 31 | gudiance | Christian Kohl | 2018 | NR | Yes | Yes | Yes | Yes | Yes | Yes | Yes | Yes | NR |
| 32 | methodological study | Ashrita Saran | 2018 | Yes | NR | Yes | Yes | Yes | NR | Yes | NR | Yes | Yes |
| 33 | gudiance | Shannon Simonovich | 2018 | Yes | NR | Yes | Yes | Yes | Yes | NR | NR | Yes | Yes |
| 34 | gudiance | Andrea C. Tricco | 2018 | NR | Yes | Yes | Yes | Yes | Yes | Yes | Yes | Yes | Yes |
| 35 | gudiance | Fares Alahdab | 2018 | Yes | Yes | Yes | Yes | Yes | NR | NR | NR | Yes | Yes |
| 36 | gudiance | Howard White | 2018 | Yes | Yes | Yes | Yes | Yes | Yes | Yes | Yes | Yes | NR |
| 37 | gudiance | Caroline Bradbury-Jones | 2019 | Yes | NR | Yes | Yes | Yes | Yes | Yes | Yes | Yes | Yes |
| 38 | gudiance | Juleen Lam | 2019 | Yes | NR | Yes | Yes | Yes | Yes | Yes | Yes | NR | Yes |
| 39 | gudiance | Taylor A.M.Wolffe | 2019 | NR | Yes | Yes | Yes | Yes | Yes | Yes | Yes | Yes | Yes |
| 40 | methodological study | 李沐阳 | 2019 | NR | NR | NR | Yes | Yes | NR | Yes | Yes | NR | Yes |
| 41 | gudiance | 田金徽 | 2019 | Yes | NR | Yes | Yes | Yes | Yes | NR | NR | NR | Yes |
| 42 | gudiance | Anthea Sutton | 2019 | NR | NR | NR | NR | NR | NR | NR | NR | NR | NR |
| 43 | gudiance | David Gough | 2019 | NR | NR | NR | NR | NR | NR | NR | NR | NR | NR |
| 44 | gudiance | Adriana Mihaela Soaita | 2019 | NR | NR | NR | Yes | Yes | Yes | NR | NR | NR | Yes |
| 45 | gudiance | Carmen Cecilia Delgado Reyes | 2019 | NR | NR | Yes | Yes | Yes | Yes | Yes | Yes | NR | Yes |
| 46 | gudiance | Benjamin E. Nye | 2020 | NR | NR | NR | NR | NR | NR | Yes | Yes | NR | Yes |
| 47 | gudiance | Howard White | 2020 | Yes | Yes | NR | Yes | Yes | Yes | Yes | Yes | Yes | Yes |
| 48 | gudiance | Daniele Wikoff | 2020 | Yes | Yes | Yes | Yes | Yes | Yes | Yes | Yes | Yes | Yes |
| 49 | methodological study | Taylor A M Wolffe | 2020 | NR | NR | NR | NR | NR | NR | NR | NR | NR | Yes |
| 50 | gudiance | 李艳飞 | 2020 | NR | NR | Yes | Yes | Yes | Yes | Yes | NR | Yes | Yes |
| 51 | gudiance | 李艳飞 | 2020 | Yes | NR | Yes | Yes | NR | NR | Yes | NR | Yes | Yes |
| 52 | gudiance | Ashrita Saran | 2020 | Yes | NR | NR | NR | NR | NR | Yes | NR | NR | NR |
| 53 | gudiance | Jon Brassey | 2021 | NR | NR | NR | NR | NR | NR | NR | NR | NR | Yes |
| 54 | gudiance | Diego Chambergo-Michilot | 2021 | NR | NR | NR | NR | NR | NR | NR | NR | NR | NR |
| 55 | gudiance | Thomas B. Røst | 2021 | NR | NR | NR | NR | NR | Yes | Yes | Yes | NR | NR |
| 56 | gudiance | Bastián Schuller-Martínez | 2021 | NR | NR | NR | NR | NR | NR | NR | NR | NR | Yes |
| 57 | gudiance | Ian Shemilt | 2021 | NR | NR | NR | NR | NR | Yes | NR | NR | NR | NR |
| 58 | gudiance | Christian A. Candela-Uribe | 2021 | NR | NR | NR | Yes | Yes | Yes | Yes | Yes | Yes | Yes |
| 59 | gudiance | Howard White | 2021 | NR | NR | NR | NR | NR | NR | NR | NR | NR | NR |
| 60 | gudiance | Hanan Khalil | 2022 | Yes | NR | Yes | Yes | Yes | NR | Yes | NR | Yes | Yes |
| 61 | gudiance | Gustavo Navas | 2022 | NR | NR | Yes | Yes | Yes | Yes | NR | NR | NR | Yes |
| 62 | gudiance | Kristina A. Thayer | 2022 | NR | NR | Yes | Yes | Yes | Yes | Yes | Yes | Yes | Yes |
| 63 | methodological study | ERNO VANHALA | 2022 | NR | NR | NR | Yes | Yes | NR | Yes | Yes | NR | NR |
| 64 | gudiance | Andrew S Pullin(CEE) | 2022 | Yes | Yes | Yes | Yes | Yes | Yes | Yes | Yes | Yes | Yes |
| 65 | gudiance | Fiona Campbell1 | 2023 | NR | NR | NR | NR | NR | NR | Yes | NR | Yes | NR |
| 66 | gudiance | Joshua R. Polanin | 2023 | NR | NR | NR | NR | NR | NR | NR | NR | NR | Yes |
| 67 | methodological study | Emily South | 2023 | NR | NR | NR | NR | NR | NR | NR | NR | NR | Yes |
| 68 | methodological study | Hanan Khalil | 2023 | Yes | Yes | Yes | Yes | NR | NR | NR | NR | NR | Yes |

*NR: Not Reported

**Supplement Table 8. Potential reporting characteristics for results of mapping reviews**

| **N.** | **Type of study** | **1st Author** | **Year** | **Study selection** | **Study characteristics** | **Risk of bias in included studies** | **Mapping analysis** |
| --- | --- | --- | --- | --- | --- | --- | --- |
| 1 | gudiance | David L. Katz | 2003 | Flow of studies: The final reports produced thus characterize the extent of pertinent evidence (plotted the number, distribution, size, and methods of studies addressing a broad content area), the overall quality of retrievable evidence, areas appropriate for qualitative or quantitative synthesis, key evidence gaps, and those methods convincingly shown to be effective or ineffective. | NR | The final reports produced thus characterize the extent of pertinent evidence (plotted the number, distribution, size, and methods of studies addressing a broad content area), the overall quality of retrievable evidence, areas appropriate for qualitative or quantitative synthesis, key evidence gaps, and those methods convincingly shown to be effective or ineffective. | Maps of included studies: A"map" of the evidence underlying CAM. Spreadsheets were created to list the num-d ber of articles retrieved in cach category of methodology pertaining to each of the condition/treatment pairs.  Areas with adequate evidence & Evidence gaps and clusters:Provided detailed analysis in priority "regions" of the map in the form of systematic review and metaanalysis; and identified those areas of the map conducive to additional systematic review (clusters). The final reports produced thus characterize the extent of pertinent evidence, the overall quality of retrievable evidence, areas appropriate for qualitative or quantitative synthesis, key evidence gaps, and those methods convincingly shown to be effective or ineffective. Reports to follow, indicating where evidenced is abundant, where scant, and where research is most needed to fill in the gaps in the evidence base. |
| 2 | gudiance | ﻿Salina Bates | 2007 | NR | NR | NR | NR |
| 3 | gudiance | ﻿Sarah E. Hetrick | 2008 | NR | NR | NR | NR |
| 4 | gudiance | ﻿Anne F. Parkhill | 2008 | NR | NR | NR | NR |
| 5 | gudiance | ﻿Maria J. Grant | 2009 | NR | NR | NR | NR |
| 6 | gudiance | Russell R | 2009 | NR | NR | NR | NR |
| 7 | gudiance | Janet Clapton (SCIE) | 2009 | Flow of studies: Standardised flow chart of literature through map | NR | ‘standard’ study quality result charts (under review) | Maps of included studies: Standardised core result charts - for example a cross tabulation of location x evaluation type to highlight countries that focus on theory vs. countries that focus on evaluated interventions (under development). Map specific result charts - these will need to be chosen on a project by project basis by the map team. |
| 8 | gudiance | ﻿Barbara A. Kitchenham | 2010 | NR | NR | NR | NR |
| 9 | gudiance | Cynthia Lum | 2010 | NR | NR | NR | NR |
| 10 | gudiance | ﻿Peter Bragge | 2011 | Flow of studies: Reporting on yield | Detailed study characteristics-The following data were extracted: study design, country, sample size, source population, interventions, outcome measures, patient factors (demographics, injury classification). This facilitated a more in-depth evidence map for the clinical question, as reflected by both the output table and the scope of the commentary on evidence | NR | Maps of included studies: Interventions and study design-Extracting to this level involved identifying the number of studies by study design for each intervention addressing the question. Where the question did not pertain to an intervention, relevant studies were listed by study design only. |
| 11 | gudiance | ﻿李 伦 | 2011 | NR | NR | NR | NR |
| 12 | methodological study | ﻿C. Schmucker | 2013 | NR | NR | NR | NR |
| 13 | gudiance | Birte Snilstveit | 2013 | NR | NR | NR | NR |
| 14 | gudiance | Madeleine C. McKinnon. | 2015 | NR | NR | NR | NR |
| 15 | gudiance | Kai Petersen | 2015 | Excluded studies: Appendix with included as well as excluded borderline papers | NR | NR | Maps of included studies: Present the outcomes of the study and structure the section with respect to the mapping questions |
| 16 | gudiance | Andrew Booth | 2015 | NR | NR | NR | NR |
| 17 | gudiance | Helen R. Bayliss | 2016 | NR | NR | NR | NR |
| 18 | gudiance | Barbara Buchberger | 2016 | NR | NR | NR | NR |
| 19 | gudiance | Diane Cooper | 2016 | NR | NR | NR | NR |
| 20 | methodological study | ﻿Neal R. Haddaway | 2016 | NR | NR | NR | NR |
| 21 | gudiance | Katy L. James | 2016 | Flow of studies: Reporting of specific details (such as search string modification for individual academic databases, search dates and numbers of results) can be documented within supplementary information. A description of the volume and characteristics of the evidence base. Excluded studies: CEE requires that the report be accompanied by a list of excluded articles assessed at full text with reasons for exclusion | NR | (Where critical appraisal is included.) A description of the evidence to include relative reliability of subsets of studies. A description of whether the evidence within each study is consistent, contested or mixed may also be included. | Maps of included studies & Evidence gaps and clusters: A description of the volume and characteristics of the evidence base, including generic (e.g. geographical location, publication source) and study-specific trends (e.g. the number and type of population and interventions studied and outcomes measured) as well as describing more complex and in depth analysis of trends in the evidence base. (Where critical appraisal is included.) A description of the evidence to include relative reliability of subsets of studies. A description of whether the evidence within each study is consistent, contested or mixed may also be included. Recommendations for primary research based on knowledge gaps that have been identified, and recommendations for secondary research in relation to knowledge clusters. Priorities and scope for future systematic review based on the available evidence and policy/practice needs. |
| 22 | methodological study | Isomi M. Miake-Lye | 2016 | NR | NR | NR | NR |
| 23 | gudiance | Eva A Rehfuess | 2016 | NR | NR | NR | NR |
| 24 | gudiance | Birte Snilstveit | 2016 | NR | NR | NR | NR |
| 25 | gudiance | Carol L. Perryman | 2016 | NR | NR | NR | NR |
| 26 | gudiance | Department of Planning, Monitoring and Evaluation (DPME) | 2016 | NR | NR | NR | NR |
| 27 | gudiance | N. R. Haddaway | 2017 | NR | NR | NR | NR |
| 28 | gudiance | Bethan C. O’Leary | 2017 | Excluded studies: Supporting tables should include a list of excluded studies with reasons for exclusion | a list of relevant reviews with their unique identifier and review score | A list of relevant reviews with their unique identifier and review score | Evidence gaps and clusters: a series of tables detailing the meta-analyses and narrative syntheses examining each refined question, designed to direct end-users to the most relevant and rigorous review for their requirements. |
| 29 | gudiance | Birte Snilstveit (3ie) | 2017 | NR | NR | NR | NR |
| 30 | gudiance | Neal R. Haddaway | 2018 | Flow of studies: Describe the review process including the volume of evidence identified from all sources and retained through each stage of the review. Must also display the number of articles/studies included at all stages of the review in a flow diagram, including the number of articles/studies excluded at each stage.  Excluded studies: Additional file containing list of and reasons for full text exclusions. | Describe the body of evidence identified using figures and tables, avoiding vote-counting (tallying of studies based on results; direction or significance). Each must be presented with descriptive information (meta-data). Describe the validity of individual studies and the evidence base as a whole (if critical appraisal conducted). | Describe the validity of individual studies and the evidence base as a whole (if critical appraisal conducted). | Maps of included studies: Systematic map database-Additional file containing meta-data and coding for included studies. Evidence gaps and clusters: Describe knowledge gaps (unrepresented or underrepresented subtopics that warrant further primary research) and knowledge clusters (well-represented subtopics that are amenable to full synthesis via systematic review) |
| 31 | gudiance | Christian Kohl | 2018 | Flow of studies: a flow diagram summarising the study selection process, satisfying PRISMA standards Excluded studies: The outcomes of the consistency check and study selection across the different stages (title, abstract and full text) including the reasons for exclusion (xlsx) | NR | The results of the critical appraisal (xlsx) | NR |
| 32 | methodological study | Ashrita Saran | 2018 | NR | NR | NR | NR |
| 33 | gudiance | Shannon Simonovich | 2018 | NR | NR | NR | NR |
| 34 | gudiance | Andrea C. Tricco | 2018 | Flow of studies: Give numbers of sources of evidence screened, assessed for eligibility, and included in the review, with reasons for exclusions at each stage, ideally using a flow diagram. | Characteristics of sources of evidence. For each source of evidence, present characteristics for which data were charted and provide the citations. | If done, present data on critical appraisal of included sources of evidence (see item 12). | Maps of included studies: For each included source of evidence, present the relevant data that were charted that relate to the review questions and objectives. Summarize and/or present the charting results as they relate to the review questions and objectives. |
| 35 | gudiance | Fares Alahdab | 2018 | NR | NR | NR | NR |
| 36 | gudiance | Howard White | 2018 | Flow of studies: Give numbers of sources of evidence screened, assessed for eligibility, and included in the review, with reasons for exclusions at each stage, ideally using a flow diagram. Excluded studies: List in the report key excluded studies (i.e., those a reader might reasonably have expected to find) and provide justification for each exclusion. | Provide details of references(M); Included studies(M); Filter for selected characteristics of included studies | All systematic reviews should be appraised for quality or confidence. Present a “risk of bias and/or study quality or confidence” table for each included study, with judgments about risks of bias, and explicit supports for these judgments.  Provide a brief narrative summary of the quality or confidence of systematic reviews in results.  Summarize the study quality/risk of bias by dimensions of the map.  Assessments of the quality of the body of evidence | Maps of included studies: EGM, Main findings in terms of spread and concentration of evidence across intervention and outcome categories highlighting important evidence gaps and trends identified in the research literature. Additional findings from filters such as study design, geographical location (ideally both regions and countries), population, confidence in study findings (assessed through standardised checklists), funding and implementing agency for the included studies. |
| 37 | gudiance | Caroline Bradbury-Jones | 2019 | NR | NR | NR | NR |
| 38 | gudiance | Juleen Lam | 2019 | NR | NR | NR | NR |
| 39 | gudiance | Taylor A.M.Wolffe | 2019 | NR | NR | NR | NR |
| 40 | methodological study | 李沐阳 | 2019 | Flow of studies: 纳入研究及证据总结情况:纳入的证据图全文纳入文献数量为 6 ～ 884 篇，其中40. 6%证据图全文纳入研究数量 ＞ 100 篇。 | NR | NR | NR |
| 41 | gudiance | 田金徽 | 2019 | Flow of studies: 文献检索结果呈现: ①根据预先制定的检索策略 和计划检索数据库所获得的检索结果以及通过其他 途径检索获得的文献数量; ②利用文献管理软件去重 后获得的文献数量; ③采用文献筛选方法，依据纳入 排除标准对去重后文献进行筛选，初步纳入符合标准 的研究，并记录排除研究的原因; ④在阅读全文基础 上，有多少个研究被排除及其原因，最终有多少个研究被纳入分析。建议使用文献筛选流程图呈现。 Excluded studies: 在阅读全文基础上，有多少个研究被排除及其原因 | 研究基本特征呈现: 推荐用表格和图( 如条状图、气泡图和热图等) 呈现纳入研究基本特征，主要为资料提取表中研究对象、干预措施和测量指标部分，但还需考虑还有那些 特征是重要的、证据使用者和患者所关注的内容。 | 纳入研究质量评价结果呈现: 对于研究质量评价结果，证据图并不一定必须呈现，已经发表的证据图多采用条形图和气泡图呈现质量评价结果。 | Maps of included studies: 总结证据时，建议采用文字和相关图表，这样有助于证据使用者了解和使用证据，目前发表的证 据图采用条形图、气泡图、森林图、雷达图、茎叶图、 折线图、饼状图、热图和散点图总结证据。 |
| 42 | gudiance | Anthea Sutton | 2019 | NR | NR | NR | NR |
| 43 | gudiance | David Gough | 2019 | NR | NR | NR | NR |
| 44 | gudiance | Adriana Mihaela Soaita | 2019 | NR | NR | NR | NR |
| 45 | gudiance | Carmen Cecilia Delgado Reyes | 2019 | NR | NR | NR | NR |
| 46 | gudiance | Benjamin E. Nye | 2020 | NR | NR | NR | NR |
| 47 | gudiance | Howard White | 2020 | NR | NR | confidence in study findings (assessed through standardised checklists) | Maps of included studies: EGM, Main findings in terms of spread and concentration of evidence across intervention and outcome categories highlighting important evidence gaps and trends identified in the research literature. Additional findings from filters such as study design, geographical location (ideally both regions and countries), population, confidence in study findings (assessed through standardised checklists), funding and implementing agency for the included studies. Areas with adequate evidence & Evidence gaps and clusters: Implications for policy and future research and key recommendations. |
| 48 | gudiance | Daniele Wikoff | 2020 | NR | NR | NR | NR |
| 49 | methodological study | Taylor A M Wolffe | 2020 | NR | NR | NR | NR |
| 50 | gudiance | 李艳飞 | 2020 | Flow of studies: 纳入研究的数量 | 纳入研究的基本特征 | 质量评价结果（系统评价） | Maps of included studies: 对研究问题特征的证据图谱展示 |
| 51 | gudiance | 李艳飞 | 2020 | NR | NR | NR | NR |
| 52 | gudiance | Ashrita Saran | 2020 | NR | NR | NR | NR |
| 53 | gudiance | Jon Brassey | 2021 | NR | NR | NR | NR |
| 54 | gudiance | Diego Chambergo-Michilot | 2021 | NR | NR | NR | NR |
| 55 | gudiance | Thomas B. Røst | 2021 | NR | NR | NR | NR |
| 56 | gudiance | Bastián Schuller-Martínez | 2021 | NR | NR | NR | NR |
| 57 | gudiance | Ian Shemilt | 2021 | NR | NR | NR | NR |
| 58 | gudiance | Christian A. Candela-Uribe | 2021 | NR | NR | NR | NR |
| 59 | gudiance | Howard White | 2021 | NR | NR | NR | NR |
| 60 | gudiance | Hanan Khalil | 2022 | NR | NR | NR | NR |
| 61 | gudiance | Gustavo Navas | 2022 | NR | NR | NR | NR |
| 62 | gudiance | Kristina A. Thayer | 2022 | Flow of studies: Provided study flow diagram examples of presenting the literature screening results, including example text that can be used when machine learning software is used. | NR | This section should describe the nature of the evidence base focusing on study design features, population or animal model system assessed, health outcomes assessed, results (optional), and study evaluation (optional). The text should be concise. Web-based interactive visualizations that provide downloadable access to the summarized content are encouraged. | Maps of included studies: This section should describe the nature of the evidence base focusing on study design features, population or animal model system assessed, health outcomes assessed, results (optional), and study evaluation (optional). The text should be concise. Web-based interactive visualizations that provide downloadable access to the summarized content are encouraged. |
| 63 | methodological study | ERNO VANHALA | 2022 | Flow of studies: Study described how the number of publications is reduced from the initial search results to the set of accepted articles. | NR | NR | NR |
| 64 | gudiance | Andrew S Pullin(CEE) | 2022 | Flow of studies: Report here the number of articles and the studies therein found in the search. A flow diagram reporting all stages of the inclusion/exclusion process, from search results to full text eligibility, should be presented. Results of consistency checking at all stages must be provided. Excluded studies: A list of studies excluded at full text together with reasons for exclusion (reasons for exclusion should match your eligibility criteria). | Review descriptive statistics (sub-headings as applicable). Descriptive statistics should be provided on any relevant information on the distribution of the articles found (e.g. geographical, temporal, institutional) in order to assess potential gaps or bias in the evidence. For full transparency, additional files are expected here including. Tables of search results showing where eligible articles were found (i.e. through which database etc) A full reference list of all eligible articles A list of studies excluded at full text together with reasons for exclusion (reasons for exclusion should match your eligibility criteria). | Mapping the quality of studies relevant to the question. The map should provide some preliminary estimate of the quality of the available evidence. This may involve providing a description of the design of each study (or of a representative sample of studies). | Maps of included studies: Mapping the quantity of studies relevant to the question. Present here a figure or a database, showing how the relevant literature is organised (categories, coding...) according to transparent, replicable criteria. This map should be readily updatable. Evidence gaps and clusters: This section should include an explanation of how the map can be used to find appropriate studies and observations on the distribution of articles and relative quantity and quality of available evidence with respect to the broad question and how the question might be broken down to enable full systematic review(s) to be conducted in future. Describe knowledge gaps (unrepresented or underrepresented subtopics that warrant further primary research) and knowledge clusters (well-represented subtopics that are amenable to full synthesis via systematic review) |
| 65 | gudiance | Fiona Campbell1 | 2023 | NR | NR | NR | NR |
| 66 | gudiance | Joshua R. Polanin | 2023 | NR | NR | NR | NR |
| 67 | methodological study | Emily South | 2023 | NR | NR | NR | NR |
| 68 | methodological study | Hanan Khalil | 2023 | Flow of studies: Number of studies-A total of 322 reviews stated the number of studies included in the mapping review (96.1%), ranging from a minimum of zero (n = 1) to a maximum of 119,546. The median was 85 studies. The remaining 13 reviews did not report the number of studies used to conduct the mapping review. Figure 8 details the number of studies included in the mapping reviews. For example, 56 mapping reviews included between 51 to 100 studies and 11 mapping reviews included more than 10,000 studies. | Equity. The majority of the mapping reviews did not address equity (n = 258; 77.0%). Equity was addressed in 66 mapping reviews (19.7%) whilst in the remaining 11 studies, it was not able to be categorically established (3.3%). Equity was defined in our review when authors described the population by either low income or racialized populations or if they have included outcome data on a particular type of population such as disadvantaged groups, or groups from a specific region that are known to be at a disadvantage, for example, sub-Saharan Africa. | Critical appraisal-A total of 87 mapping reviews performed a critical appraisal of the evidence and reported the findings in the study (26.0%). The majority of mapping reviews (n = 248) did not present the findings of a critical appraisal (74.0%). | Maps of included studies: The majority of the mapping reviews (n = 322; 96.1%) presented accompanying graphics or visuals to communicate findings. Equity. The majority of the mapping reviews did not address equity (n = 258; 77.0%). Equity was addressed in 66 mapping reviews (19.7%) whilst in the remaining 11 studies, it was not able to be categorically established (3.3%). Equity was defined in our review when authors described the population by either low income or racialized populations or if they have included outcome data on a particular type of population such as disadvantaged groups, or groups from a specific region that are known to be at a disadvantage, for example, sub-Saharan Africa, and other underdeveloped countries. |

*NR: Not Reported

**Supplement Table 9. Potential reporting characteristics for discussion, conclusions, and other items of mapping reviews**

| **N.** | **Type of study** | **1st Author** | **Year** | **Discussion** | **Conclusions** | **Acknowledgements** | **Contributions of authors** | **Declarations of interest** | **Sources of support** |
| --- | --- | --- | --- | --- | --- | --- | --- | --- | --- |
| 1 | gudiance | David L. Katz | 2003 | Plans for map updates: As with any maps, evidence maps require updating at reasonable intervals to keep pace with a changing landscape. | NR | NR | NR | NR | NR |
| 2 | gudiance | ﻿Salina Bates | 2007 | NR | NR | NR | NR | NR | NR |
| 3 | gudiance | ﻿Sarah E. Hetrick | 2008 | NR | NR | NR | NR | NR | NR |
| 4 | gudiance | ﻿Anne F. Parkhill | 2008 | NR | NR | NR | NR | NR | NR |
| 5 | gudiance | ﻿Maria J. Grant | 2009 | NR | NR | NR | NR | NR | NR |
| 6 | gudiance | Russell R | 2009 | NR | NR | NR | NR | NR | NR |
| 7 | gudiance | Janet Clapton (SCIE) | 2009 | Limitations of the review: Non standardised, map specific Plans for map updates | NR | NR | NR | NR | NR |
| 8 | gudiance | ﻿Barbara A. Kitchenham | 2010 | NR | NR | NR | NR | NR | NR |
| 9 | gudiance | Cynthia Lum | 2010 | Plans for map updates | NR | NR | NR | NR | NR |
| 10 | gudiance | ﻿Peter Bragge | 2011 | Plans for map updates | NR | NR | NR | NR | NR |
| 11 | gudiance | ﻿李 伦 | 2011 | NR | NR | NR | NR | NR | NR |
| 12 | methodological study | ﻿C. Schmucker | 2013 | NR | NR | NR | NR | NR | NR |
| 13 | gudiance | Birte Snilstveit | 2013 | Plans for map updates: It is desirable to have a maintenance plan to update maps annually | NR | NR | NR | NR | NR |
| 14 | gudiance | Madeleine C. McKinnon. | 2015 | NR | NR | NR | NR | NR | NR |
| 15 | gudiance | Kai Petersen | 2015 | Limitations of the review | NR | NR | NR | NR | NR |
| 16 | gudiance | Andrew Booth | 2015 | NR | NR | NR | NR | NR | NR |
| 17 | gudiance | Helen R. Bayliss | 2016 | Plans for map updates | NR | NR | NR | NR | NR |
| 18 | gudiance | Barbara Buchberger | 2016 | NR | NR | NR | NR | NR | NR |
| 19 | gudiance | Diane Cooper | 2016 | NR | NR | NR | NR | NR | NR |
| 20 | methodological study | ﻿Neal R. Haddaway | 2016 | NR | NR | NR | NR | NR | NR |
| 21 | gudiance | Katy L. James | 2016 | Priorities and scope for future systematic review based on the available evidence and policy/practice needs. Implications for research, policy and practice. Plans for map updates | NR | NR | NR | NR | NR |
| 22 | methodological study | Isomi M. Miake-Lye | 2016 | NR | NR | NR | NR | NR | NR |
| 23 | gudiance | Eva A Rehfuess | 2016 | NR | NR | NR | NR | NR | NR |
| 24 | gudiance | Birte Snilstveit | 2016 | Plans for map updates | NR | NR | NR | NR | NR |
| 25 | gudiance | Carol L. Perryman | 2016 | Plans for map updates | NR | NR | NR | NR | NR |
| 26 | gudiance | Department of Planning, Monitoring and Evaluation (DPME) | 2016 | Plans for map updates | NR | NR | NR | NR | NR |
| 27 | gudiance | N. R. Haddaway | 2017 | NR | NR | NR | NR | NR | NR |
| 28 | gudiance | Bethan C. O’Leary | 2017 | NR | NR | NR | NR | NR | NR |
| 29 | gudiance | Birte Snilstveit (3ie) | 2017 | NR | NR | NR | NR | NR | NR |
| 30 | gudiance | Neal R. Haddaway | 2018 | Limitations of the review Implications Plans for map updates | Conclusions | NR | NR | Declarations of interest | NR |
| 31 | gudiance | Christian Kohl | 2018 | NR | NR | NR | NR | NR | NR |
| 32 | methodological study | Ashrita Saran | 2018 | Plans for map updates | NR | NR | NR | NR | NR |
| 33 | gudiance | Shannon Simonovich | 2018 | NR | NR | NR | NR | NR | NR |
| 34 | gudiance | Andrea C. Tricco | 2018 | Summary of main results Limitations of the review | Conclusions | NR | NR | NR | Sources of support |
| 35 | gudiance | Fares Alahdab | 2018 | NR | NR | NR | NR | NR | NR |
| 36 | gudiance | Howard White | 2018 | Summary of main results Limitations of the review Implications Plans for map updates | Conclusions | Acknowledgements | Contributions of authors | Declarations of interest | Sources of support |
| 37 | gudiance | Caroline Bradbury-Jones | 2019 | NR | NR | NR | NR | NR | NR |
| 38 | gudiance | Juleen Lam | 2019 | NR | NR | NR | NR | NR | NR |
| 39 | gudiance | Taylor A.M.Wolffe | 2019 | Plans for map updates | NR | NR | NR | NR | NR |
| 40 | methodological study | 李沐阳 | 2019 | NR | NR | NR | NR | NR | NR |
| 41 | gudiance | 田金徽 | 2019 | NR | NR | NR | NR | NR | NR |
| 42 | gudiance | Anthea Sutton | 2019 | NR | NR | NR | NR | NR | NR |
| 43 | gudiance | David Gough | 2019 | NR | NR | NR | NR | NR | NR |
| 44 | gudiance | Adriana Mihaela Soaita | 2019 | NR | NR | NR | NR | NR | NR |
| 45 | gudiance | Carmen Cecilia Delgado Reyes | 2019 | NR | NR | NR | NR | NR | NR |
| 46 | gudiance | Benjamin E. Nye | 2020 | NR | NR | NR | NR | NR | NR |
| 47 | gudiance | Howard White | 2020 | Implications Plans for map updates | NR | NR | NR | NR | NR |
| 48 | gudiance | Daniele Wikoff | 2020 | Plans for map updates | NR | NR | NR | NR | NR |
| 49 | methodological study | Taylor A M Wolffe | 2020 | NR | NR | NR | NR | NR | NR |
| 50 | gudiance | 李艳飞 | 2020 | 研究报告的撰写结构证据图谱研究报告的撰写结构与系统评价/Meta 分析相似，一般包括研究背景、材料和方法 （研究设计、纳入排除标准、检索策略、数据提取及 文献筛选、质量评价、数据分析）、结果（纳入研究的数量及基本特征、质量评价结果、证据图谱的展示）、讨论、结论及参考文献 | 结论 | NR | NR | NR | NR |
| 51 | gudiance | 李艳飞 | 2020 | NR | NR | NR | NR | NR | NR |
| 52 | gudiance | Ashrita Saran | 2020 | Plans for map updates: Gap maps should be updated regularly, at least every two years, to ensure the latest evidence is included. | NR | NR | NR | NR | NR |
| 53 | gudiance | Jon Brassey | 2021 | NR | NR | NR | NR | NR | NR |
| 54 | gudiance | Diego Chambergo-Michilot | 2021 | NR | NR | NR | NR | NR | NR |
| 55 | gudiance | Thomas B. Røst | 2021 | NR | NR | NR | NR | NR | NR |
| 56 | gudiance | Bastián Schuller-Martínez | 2021 | NR | NR | NR | NR | NR | NR |
| 57 | gudiance | Ian Shemilt | 2021 | Plans for map updates | NR | NR | NR | NR | NR |
| 58 | gudiance | Christian A. Candela-Uribe | 2021 | NR | NR | NR | NR | NR | NR |
| 59 | gudiance | Howard White | 2021 | NR | NR | NR | NR | NR | NR |
| 60 | gudiance | Hanan Khalil | 2022 | Plans for map updates | NR | NR | NR | NR | NR |
| 61 | gudiance | Gustavo Navas | 2022 | NR | NR | NR | NR | NR | NR |
| 62 | gudiance | Kristina A. Thayer | 2022 | Plans for map updates | Conclusions | NR | NR | NR | NR |
| 63 | methodological study | ERNO VANHALA | 2022 | NR | NR | NR | NR | NR | NR |
| 64 | gudiance | Andrew S Pullin(CEE) | 2022 | Limitations of the review:A detailed and reflective discussion of the limitations of the review is expected here, including limitations due to the search strategy (limitations of the review methods), as well as limitations due to underlying bias within the studies found such as baseline bias and confounding variables (limitations of the evidence base). Please do not provide a discussion section that includes speculation or expert opinion concerning the review findings. Plans for map updates | Implication for Policy/Management:This section summarises the state of the evidence base in terms of the distribution and abundance of studies captured in the map in relation to different elements of the question. Potential for unpacking the broad question and enabling more detailed evidence synthesis should be highlighted. The intention is to inform and any form of advocacy should be excluded. Implication for Research:This section summarises the shortcomings of the current evidence base in terms of knowledge gaps and the need for primary research. In this section some advocacy for research is permissible provided it is clearly justified by the review outcome. This should take the form of recommendations for future study designs that would improve the evidence base. | Acknowledgements | Contributions of authors | Declarations of interest | Sources of support |
| 65 | gudiance | Fiona Campbell1 | 2023 | NR | NR | NR | NR | NR | NR |
| 66 | gudiance | Joshua R. Polanin | 2023 | NR | NR | NR | NR | NR | NR |
| 67 | methodological study | Emily South | 2023 | NR | NR | NR | NR | NR | NR |
| 68 | methodological study | Hanan Khalil | 2023 | Limitations of the review Methodological challenges | NR | NR | NR | NR | Sources of support |

*NR: Not Reported
